# Malignant Airway Obstruction and Endobronchial Stent Placement: A Systematic Review on the Efficacy and Safety

**DOI:** 10.7759/cureus.40912

**Published:** 2023-06-24

**Authors:** Zaryab Umar, Muhammad Haseeb ul Rasool, Asma U Hosna, Avish Parikh, Jonathan Ariyaratnam, Jasmine K Sandhu, Salman Ashfaq, Nazaakat Ahmed, Jawad Khan, Theo Trandafirescu

**Affiliations:** 1 Internal Medicine, Icahn School of Medicine at Mount Sinai, Queens Hospital Center, New York, USA; 2 Medicine, Icahn School of Medicine at Mount Sinai, Queens Hospital Center, New York, USA; 3 Medicine, Queens Hospital Center, New York, USA; 4 Medicine, Allama Iqbal Medical College, Lahore, PAK; 5 Internal Medicine, Queens Hospital Center, New York, USA

**Keywords:** endotracheal stent, metallic stent, silicone stent, self-expanding metal stents, endobronchial stent, malignant airway obstruction

## Abstract

The systematic review aims to evaluate the efficacy and safety of endobronchial stent placement for malignant airway obstruction.

A comprehensive search was conducted across multiple databases to identify relevant studies. Cohort studies, randomized controlled trials, and case-control studies examining the outcomes of endobronchial stent placement in patients with malignant airway obstruction were included. Data on pre-treatment evaluation, such as pulmonary function testing, dyspnea severity scoring systems, arterial blood gas parameters, imaging, and degree of obstruction, were also collected. Primary outcomes of interest included post-procedure stenosis, pulmonary function testing evaluation, blood gas parameters, and survival outcomes. Secondary outcomes encompassed improvements in clinical status, dyspnea grade, and procedure-related complications.

A total of 27 studies met the inclusion criteria and were included in the systematic review. The included studies demonstrated promising outcomes of endobronchial stent placement in managing malignant airway obstruction. Post-procedure airway diameters, pulmonary function testing, and blood gas parameters improved significantly. Survival outcomes varied among studies. Furthermore, endobronchial stent placement was associated with improvements in clinical status and dyspnea grade. Procedure-related complications ranged from pain, hemoptysis and mucus plugging to stent obstruction, migration and pneumothorax.

This systematic review suggests that endobronchial stent placement is an effective and safe intervention for managing malignant airway obstruction. It offers significant improvements in post-procedure stenosis, pulmonary function testing, blood gas parameters, and clinical outcomes. However, further studies with larger sample sizes and standardized reporting are warranted to better evaluate the long-term efficacy and safety of endobronchial stent placement for malignant airway obstruction.

## Introduction and background

Central airway obstruction (CAO) is a complex problem mainly secondary to malignant lesions and to some extent, due to benign lesions. Central airway obstruction can be caused by extrinsic mass compression, intrinsic exophytic tumor, or dynamic collapse. Malignant CAO may be caused by primary lung or esophageal cancer but also by metastatic cancer leading to mass in the thoracic cavity [1]. Bronchogenic carcinoma is the most common cause of malignant CAO. CAO increases the risk of post-obstructive pneumonia and respiratory failure. Around 30% of lung cancer patients develop CAO. Unfortunately, the development of CAO decreases the survival rate remarkably; if CAO is untreated, survival is usually two to three months, but with interventional treatment survival rate improves to six to eight months [2,3]. 

Endoscopic management can be an essential addition to existing treatment options for symptomatic tracheobronchial complications in unresectable benign or malignant airway obstruction cases. Various endoscopic interventions are available to treat malignant CAO, including endobronchial dilation, laser therapy, and airway stents. These procedures provide symptomatic relief and improve quality of life [1,4]. The symptoms-free survival rate has increased significantly over the last few decades because of technical advances in interventional bronchoscopy procedures [5].

Stent insertion is recommended for extrinsic compression causing airway obstruction. Various stent models have been developed for the treatment of inoperable stenoses of the airway [1]. As the number of patients requiring stent placement is increasing day by day, an in-depth discussion of the topic is much needed. Here we present a systematic review of endobronchial stent placement as well as some practical issues related to airway stents.

## Review

Materials and methods

Search Strategy and Study Selection

This study followed Preferred Reporting Items for Systematic Reviews and Meta-Analyses (PRISMA) guidelines for systematic reviews and meta-analyses, which do not require protocol registration [6]. An electronic database search was conducted for relevant studies published from 12/31/2022 to 04/02/2023 on PubMed, Embase, and Cochrane using certain keywords. Table 1 provides a detail of the search terms used on PubMed, Cochrane, and Embase along with the results obtained.

**Table 1:** Search strategy used for each database. MeSH: Medical Subject Headings

Database	Search strategy	Results
PubMed	Bronchoscopy[tw] OR Bronch*[tw] OR "Bronchoscopy"[Mesh] AND Airway stent[tw] OR Stent[tw] OR "Stents"[Mesh] OR "Catheterization"[Mesh] AND Airway[tw] OR Obstruction[tw] OR “Bronchial obstruction” [tw] OR “Tracheobronchial obstruction” [tw] OR "Airway Obstruction"[Mesh] AND “Lung cancer” [tw] OR Cancer[tw] OR Neoplasm[tw] OR Malig*[tw] OR Malignancy[tw] OR "Lung Neoplasms"[Mesh] OR "Neoplasms"[Mesh]	376
Cochrane	Bronchoscopy AND Stent OR Catheterization OR Airway stent AND Tracheobronchial obstruction OR Bronchial obstruction OR Airway obstruction AND Lung neoplasm OR Lung malignancy OR Lung cancer OR Malignancy OR Tumor OR Cancer	1
Embase	Fiberoptic bronchoscopy OR Bronchoscopy AND Tracheobronchial stent AND Airway obstruction OR Bronchus obstruction OR Trachea obstruction OR Trachea stenosis AND Lung cancer OR Lung tumor OR Neoplasm OR Malignant neoplasm OR Malignant	5

We conducted a comprehensive search to include various types of original studies (cohort, cross-sectional, randomized controlled trials and case-control) that examined the characteristics of patients with airway obstruction in the context of malignancy. We also aimed to gather information on pre-treatment evaluation, such as pulmonary function testing, dyspnea severity scoring systems, arterial blood gas parameters, imaging, and degree of obstruction. Additionally, we sought to explore outcomes and complications associated with the use of endobronchial stents in these patients. We included commentaries and case series with a minimum of 10 patients, prioritizing studies that provided sufficient data relevant to our study design. Exclusion criteria encompassed non-original reports, reviews, letters to editors, case reports or series with fewer than 10 patients, articles lacking extractable or pertinent data, non-English publications, duplicate records, animal studies, overlapping data, and inaccessible or irrelevant full texts.

Our primary outcomes of interest focused on post-procedure stenosis, pulmonary function testing evaluation, and blood gas parameters after the intervention. Survival outcomes were also a primary focus. As for secondary outcomes, we examined improvements in clinical status following laser treatment, as well as enhancements in dyspnea grade, additional scoring systems, and scales post-procedure. We also assessed procedure-related complications as a secondary outcome. To ensure comprehensive inclusion, we manually searched the reference lists of the included papers. The screening process involved two independent reviewers who assessed titles and abstracts, followed by a thorough full-text screening to ensure the inclusion of relevant papers. Any disagreements were resolved through discussion and, if necessary, by consulting the senior author.

Data Extraction

We developed a data extraction sheet using Microsoft Excel (Microsoft® Corp., Redmond, WA, USA). Two independent reviewers extracted data using the Excel sheet. Disagreements and discrepancies were resolved through discussions with the senior author.

Quality Assessment

The risk of bias in the included studies was evaluated by one independent reviewer. A risk-of-bias assessment tool developed by the National Institutes of Health (NIH) was used to assess the quality of the included studies [7]. 

Results

Search Results

**Figure 1:**
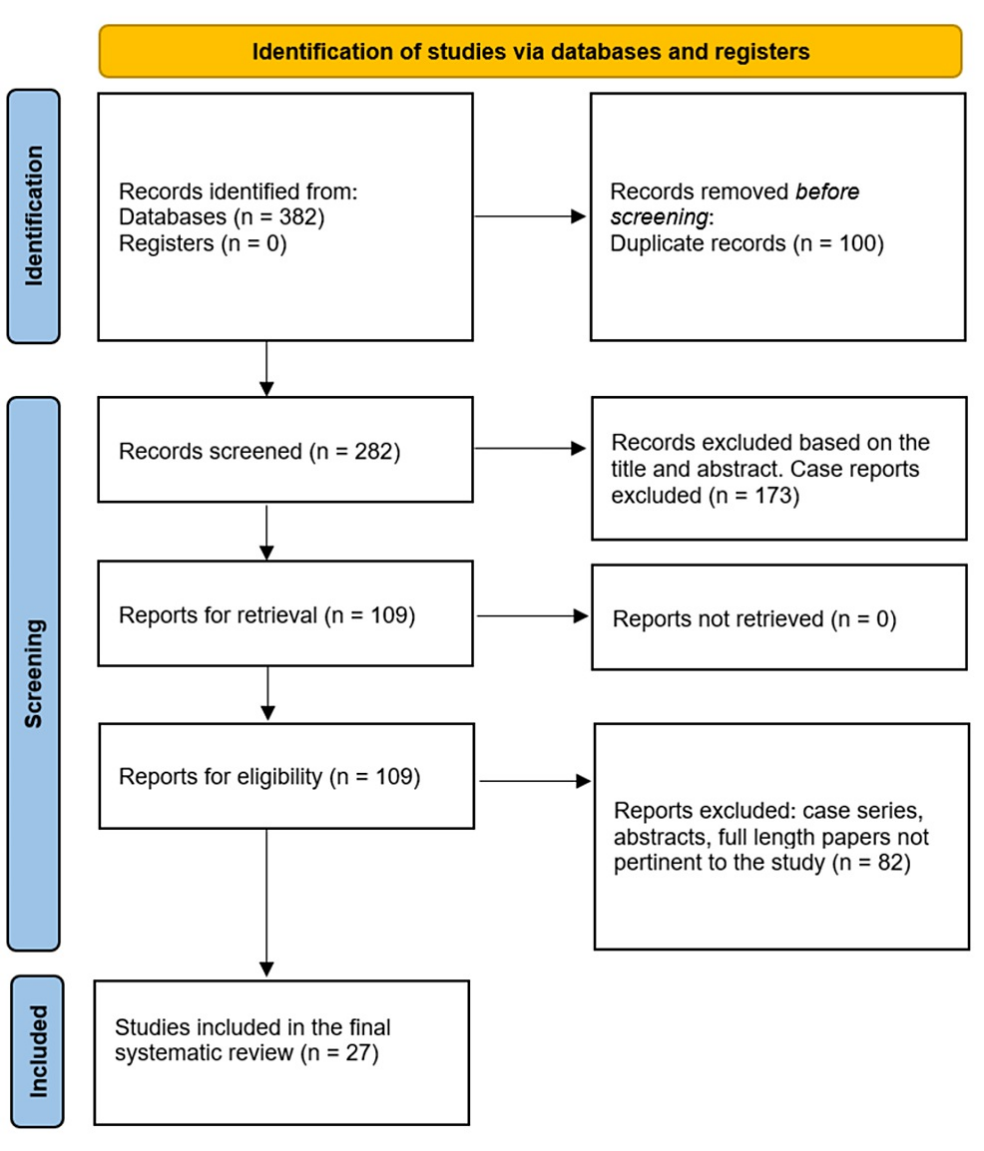
PRISMA flow diagram depicting the screening process for this systematic review and meta-analysis. PRISMA: Preferred Reporting Items for Systematic Reviews and Meta-Analyses

In our systematic review, the initial search across various databases yielded a total of 382 records. Following the removal of duplicate records, 100 were eliminated, resulting in 282 records for further screening. From this pool, 173 records were excluded based on a preliminary assessment of the title and abstract, specifically targeting case reports. The remaining 109 records were sought for full retrieval. Fortunately, all 109 reports were successfully obtained and assessed for eligibility. Among these reports, 82 were excluded due to their classification as case series, abstracts, or full-length papers deemed irrelevant to the study. Ultimately, our systematic review included a total of 27 studies, which met the eligibility criteria and were included in the final analysis. Figure 1 below provides a diagrammatic representation of the search results. 

Baseline Patient Characteristics

Table 2 provides a summary of the baseline patient characteristics.

**Table 2:** Baseline patient characteristics IQR: Interquartile range SD: Standard deviation MRC: Medical Research Council MMRC: Modified Medical Research Council

	Authors	Study design	Year	Age in years	Total number of patients (n. number of patients) (male/female)	Type of cancer (n, number of patients)/stage	Clinical presentation (n, number of patients)/characteristics of patients included in the study	Smoking status (n, number of patients)	Comorbid conditions
1.	Dalar L et al [8].	Retrospective cohort study	2016	Median age 63	547 (432/115)	Squamous cell cancer, Small cell cancer (53), Adenocarcinoma (31), Non-small cell cancer (181), Carcinoid tumor (9), Thyroid cancer (4), Renal cell cancer (4), Malignant Mesothelioma (2), Neuroendocrine tumor (9), Malignant Mesenchymal tumor (9), other metastasis (17).	Not mentioned	Smoker (446), Nonsmoker (101)	Not mentioned
2.	Dutau H et al [9].	Prospective clinical trial	2020	Mean age (Mean ± SD) 64.5 ± 10.6 years.	78 (64/14)	Squamous cell carcinoma was the main histology (45 patients).	Patients with mechanical airway obstruction due to Non-small cell lung cancer, undergoing therapeutic bronchoscopy were included, if at the end of the procedure they fulfilled following criteria for stent placement. (1) Airway lumen > 50%; (2) Absence of extrinsic compression requiring stenting; (3) Residual tumor that could entirely be covered with one straight or Y silicone stent (Tracheobronxane, Novatech) from the proximal to the distal margin.	Not mentioned	Not mentioned
3.	Grosu HB et al [10].	Retrospective cohort study	2013	Mean age (Mean ± SD) 59.4 ± 13.7.	72 (45/27)	Non-small cell lung cancer (32), Renal cell (10), Sarcoma (6), Colon (4), Thyroid (4), Melanoma (3), Breast (2), Head and neck (1), Small cell (1), Lymphoma (1), Other solid tumors metastatic to lung (8).	For patients with malignant central airway obstruction, stents were placed if there was pure extrinsic compression with 50% airway occlusion, or adequate airway patency (50%) could not be achieved with ablative techniques alone, or if it was felt that airway re-occlusion would occur quickly if a stent was not placed following ablation for a mixed obstruction.	Smokers: 5 active, 44 ex- smokers Nonsmokers: 23	Not mentioned
4.	Huang S et al [11].	Retrospective cohort study	2017	Number of patients less than 50 years of age: 3. Number of patients more than 50 years of age: 53.	45/11/56	Lung cancer (29), Esophageal cancer (27) Pathology: Others and unknown: 17, Squamous carcinoma 35, Adenocarcinoma 4.	Dyspnea, Airway Neoplasm, Extrinsic compression, Fistula.	Smokers: 24 Non-smokers: 23	Not mentioned
5.	Iyoda A et al [12].	Retrospective chart review	2021	Mean age 64, Mean age in the silicone stent (SS) group: 62 years, mean age in the metallic stent (MS) group: 65 years.	106 (80/26) SS: 33/12, MS: 47/14)	Lung cancer (52) (SS: 23 and MS: 29), Esophageal cancer (39) (SS: 14 and MS: 25), Other (15) (SS: 8 and MS: 7). Primary cancer 77 (SS: 33, MS: 44) Recurrent cancer 27 (SS: 11, MS: 16).	106 patients with central airway stenosis or obstruction due to thoracic malignancy who underwent first placement of either SS or MS at Toho University Omori Medical Center between 1998 and 2018.	Not mentioned	Not mentioned
6.	Lachkara S et al [13].	Retrospective chart review	2020	Mean age (Range) with range: Silicone Y stent (SYS): 60.6 (38-85) SEM Y stent (SEMYS: 57.7 (37-78).	SYS: 25/15 SEMYS: 28/10	SYS: Lung cancer (25), Esophageal cancer (11), Other (3), NA (1) SEMYS: Lung cancer (22), Esophageal cancer (14).	Patients with malignant carina involvement (stenosis or tracheobronchial esophageal fistula), not suitable for curative surgery, and treated with bronchial Y stent, were selected for the present study.	Not mentioned	The main indication for stenting was tracheobronchial obstruction in 61 patients, including 20 compressions (7 in the SEM Y group, 13 in the SYS group), association of tracheoesophageal fistula and obstruction in 4 patients (2 in SEM Y group), and fistula without obstruction in 13 (10 in the SEM Y group). Of the 78 patients, 25 had esophageal cancer (14 in the SEM Y group), 47 had primary lung cancer (22 in the SEM Y group), and 6 had extra thoracic primary cancer (2 in the SEM Y group).
7.	Ma G et al [14].	Retrospective chart review	2008	Median age (IQR) 57, (37-75)	52 (32/20)	30 cases caused by Lung cancer; 13 cases by Esophageal carcinoma, six cases by Lymphoma and three cases with unknown pathology.	Large airway stenosis with severe respiratory difficulties caused by malignant tumor compression or invasion.	Not mentioned	Not mentioned
8.	Marchese R et al [15].	Retrospective chart review	2015	Mean age (Mean ± SD) 64±11	51 (37/14)	Lung cancer (44) of which: Non-small cell lung cancer (18), Squamous cell cancer (19), Small cell lung cancer (7). Metastasis (5): Colon cancer (1) Esophageal cancer (1) Endometrial cancer (2) laryngeal cancer. Hemangiopericytoma (1).	All patients were symptomatic; most of them complained of dyspnea (29) of moderate degree [modified MRC (Mean ± SD) 2.6 ± 0.8} and cough (25).	Not mentioned	Not mentioned
9.	Marchese R et al [16].	Retrospective chart review	2020	Mean age 67, range 45–85 years.	51 (41/10)	51 patients affected by advanced unresectable lung cancer involving lobar bronchi and distal carina (RC1, RC2, or LC2).	Symptoms: Dyspnea (27), MMRC (Mean: 2.4 ± 0.7), Cough (22), Hemoptysis (7), Chest pain (5).	Smokers: (before stenting/ after stenting) (28/12) Ex-smoker (15/31) Never smoke (8/8).	Not mentioned
10.	Marchioni A et al [17].	Multicentric retrospective study	2020	Age (IQR): For the complete study, 74 (68-79.3). For the integrated treatment, 73.3 (66.3-78.4). For the standard treatment: 76 (71-80.5).	Total number of patients, 100. Integrated treatment, 60 patients. Standard treatment, 40 patients. Male (68). 37 in the integrated treatment group, 31 in the standard treatment group.	NSCLC/Stage IIIB	Central airway obstruction	Not mentioned	Not mentioned
11.	Miyazawa T et al [18].	Prospective multicenter study	2000	Mean: 63.0, range: 41–82 years	34(30/4)	Bronchogenic carcinoma Squamous cell carcinoma 11 Adenocarcinoma 8 Small cell carcinoma 5 Adenoid cystic carcinoma 1 Esophageal carcinoma 4 Mediastinal tumor 3 Metastatic pulmonary disease 2.	Not mentioned	Not mentioned	Not mentioned
12.	Miyazawa T et al [19].	Prospective case control	2004	Mean ± SD. Tracheal stenosis: 61.2 ± 5.9. Carinal stenosis: 64.9 ± 5.6. Bronchial stenosis: 67.7 ± 5.5. Extensive stenosis: 66.4 ± 3.0.	Tracheal stenosis: 20 (18/2) Carinal stenosis: 16 (11/5) Bronchial stenosis: 18(14/4) Extensive stenosis: 10 (7/3)	38 patients with adenocarcinoma, 18 with squamous cell carcinoma, and 8 with recurrence of small cell carcinoma after chemotherapy and/or radiotherapy/stage IIIB/IV.	Patients with World Health Organization Dyspnea Grade III–IV, stage IIIB/IV inoperable lung cancer without further treatment options, central airway stenosis due to extrinsic compression, and residual stenosis of more than 50% after balloon dilatation.	Not mentioned	Not mentioned
13.	Monnier P et al [20].	Prospective Trial	1996	Average age was 62 years (range, 36 to 83 years).	40 (29/11)	Primary tracheal or bronchial squamous cell carcinoma in 25 cases (in 3 cases there was tumoral recurrence of anaplastic small cell carcinoma after chemotherapy). 3 cases presented with esophageal squamous cell carcinoma and a tracheobronchial fistula, and other histologic features were present in 9 cases.	Severely debilitated, presenting with dyspnea and/or pulmonary or lobar atelectasis. Most of them had already undergone one or more treatments: Radiotherapy (15), pulmonary resections (11), palliative laser dilatation (10), chemotherapy (7), insertion of another stent (4).	Not mentioned	Not mentioned
14.	Nakajima Y et al [21].	Retrospective review	1999	Mean 62 years, range 52 to 83 years	22 patients (18 men, 4 women).	Bronchogenic carcinoma (14), Esophageal carcinoma (7) and Thyroid carcinoma (1). The causes of airway stenosis were extrinsic compression by mediastinal adenopathy in 17 patients and intrinsic mucosal lesions of bronchogenic carcinoma in five.	Not mentioned	Not mentioned	Not mentioned
15.	Oki M et al [22].	Retrospective chart review	2017	Median age (IQR) 62.5 years (13–86)	30 (24/4)	Lung cancer (17), Squamous cell carcinoma (10), Adenocarcinoma (6), Small cell carcinoma (1), Esophageal cancer (6), Thyroid cancer (2), Renal cell carcinoma (1), Adenoid cystic carcinoma (1), Malignant lymphoma (1), Tracheal cancer, Squamous cell carcinoma (1), Ewing sarcoma (1).	Patients with malignant airway stenosis requiring emergency intubation prior to stenting procedures.	Not mentioned	Not mentioned
16.	Özdemir C et al [23].	Retrospective chart review	2016	Mean ± SD, 58.14 ± 8.48 years. Range 44–72 years.	14 (12/2)	Non-small cell lung cancer (11), Small cell lung cancer (1).	Patients inducted into the study if airway patency was <50 % after rigid bronchoscopy intervention (dilatation and/or de-obstruction), or if the recurrence risk was high. Another indication for stent application was to cover fistula when a fistula stoma was detected by bronchoscopy evaluation in between central airway and esophagus or mediastinum.	Not mentioned	Not mentioned
17.	Razi SS et al [24].	Retrospective chart review	2010	Mean ± SD, 66 ± 13 years. Range, 44–89 years.	50 (29/21)	Non-small cell lung cancer (38), Small cell lung cancer (4), Esophageal cancer (4), Mediastinal sarcoma (2), and Metastatic colon and breast cancer (2). Nine patients had stage IIIa/b disease while 41 patients had stage IV disease at the time of initial airway intervention.	Symptomatic malignant central airway obstruction who underwent airway stenting with or without endoscopic tumor resection. Dyspnea (46), Cough (24), Chest pain (11), Hemoptysis (11) Miscellaneous (25).	Smokers (13) Past smoker (32) Never smoker (5)	Not mentioned
18.	Righini C et al [25].	Retrospective chart review	2010	Mean age (Mean ± SD) 61.7±14.0	69 (40/29)	Tracheobronchial cancers (32), Esophageal cancer (19), Thyroid cancer (9), Mediastinal malignancy (6), Other malignancies (3. 1 of each: Pulmonary sarcoma, Distant metastasis from Malignant melanoma, and Endometrial carcinoma).	Patients recorded in our hospital pharmacy order database as having an airway stent insertion for malignant airway obstruction were reviewed.	Not mentioned	Not mentioned
19.	Saji H et al [26].	Retrospective chart review	2010	Range from 42 to 91 years with a mean age of 63.9 years	59 (51/8)	Squamous cell carcinoma (30), Adenocarcinoma (20), Large cell carcinoma (4), Small cell carcinoma (3), unclassified carcinoma (2).	Not mentioned	Not mentioned	Not mentioned
20.	Tayama K et al [27].	Retrospective chart review	1997	Range 37 to 77 years (Mean age 59 years)	20 (08/12)	Esophageal carcinoma (11), Primary lung carcinoma (3), Malignant lymphoma (1), Metastatic lung carcinoma (1), Thyroid carcinoma (1), Adenoid cystic carcinoma (1), Recurrent lung carcinoma (1), Recurrent thyroid carcinoma (1).	Malignant airway obstruction	Not mentioned	Not mentioned
21.	Verma A et al [28].	Retrospective review	2018	Median (range). Total, 63 (23-86). Laser group, 63 (23-86). Ultraflex stent, 63 942-86).	30 (22/8)	Squamous cell carcinoma (5), Local extension: Lung cancer (12), Esophageal cancer (10) Anaplastic thyroid carcinoma (2), LELC (lymphoepithelial carcinoma) (1), Sarcomatoid tumor (1), Neuroendocrine cancer (1), Unidentified (1).	Malignant airway obstruction	Not mentioned	Not mentioned
22.	Wilson GE et al [29].	Retrospective chart review	1996	Mean age 64 years (range 30-82).	56(33/23)	Primary tumor type (47), Squamous cell carcinoma (25), Non-small cell cancer (3), Small cell cancer (6), Adenocarcinoma (4), Adeno-squamous carcinoma (1), Presumed carcinoma (9) Secondary tumor (9) Esophageal cancer (3), Breast cancer (2), Thyroid cancer (2), Melanoma (1), Colon cancer (1).	Respiratory distress due to malignant obstruction of the trachea and/or a main bronchus.	Not mentioned	Not mentioned
23.	Yerushalmi R et al [30].	Retrospective chart review	2006	Range 36 to 85 years (median 68).	34	Thirty-five percent of the patients had primary lung cancer and 65% had metastatic disease.	Dyspnea (82%), cough (11.7%), hemoptysis (9%), pneumonia (5.9%), and atelectasis (3%).	Not mentioned	Not mentioned
24.	Zwischenberger JB et al [31].	Retrospective chart review	1997	Range 38 to 76 years (Mean age 58 years).	14(07/07)	Poorly differentiated non-small cell cancer (6), Adenocarcinoma (2), Squamous cell carcinoma (4), Large cell cancer (1), Small cell cancer (1).	Severe dyspnea (American Thoracic Society grade 4).	Not mentioned	Not mentioned
25.	Akram MJ et al [32].	Retrospective cross-sectional review	2020	Mean age 46.63 ± 16.02.	51 (24/27)	Esophageal cancer (37), Lung cancer (6), Osteosarcoma (2), Hodgkins’s disease (1), Breast carcinoma (1), Rectal carcinoma (1), Mixed germ cell tumor (1), Sarcomatoid mediastinal cancer (1), Thyroid cancer (1).	Poor performance status (96.1%), shortness of breath (39.2%), fever with productive cough (23.5%) and stridor with shortness of breath (21.6%).	Not mentioned	Not mentioned
26.	Bolliger CT et al [33].	Prospective study	2004	Mean age 62 years (range: 37–83)	26 (16/10)	Bronchogenic carcinoma (18), Esophageal carcinoma (4), Metastases (2), Tracheal carcinoma (1), Schwannoma (1).	Dyspnea, infection, cough, hemoptysis.	Not mentioned	Not mentioned
27.	Chhajed PN et al [34].	Retrospective Study	2010	Median, 63 years	Patients in which stents were placed: 93 out of total 130 (88/42)	Values given as in number of procedures (total procedures were 167) in 130 patients as total: Lung cancer: (103), Esophageal cancer (9), pulmonary metastases (55).	Not mentioned	Not mentioned	Not mentioned

Dalar et al. (2016) conducted a retrospective cohort study involving 547 cancer patients (median age: 63) comprising various types of cancer. The study included 432 males and 115 females [8]. Dutau et al. (2020) conducted a prospective clinical trial with 78 patients (mean age: 64.5) diagnosed with squamous cell carcinoma and airway obstruction due to non-small cell lung cancer. Among them, 64 were males and 14 were females [9]. Grosu et al. (2013) conducted a retrospective cohort study involving 72 cancer patients (mean age: 59.4) with different types of cancer. The study included 45 males and 27 females [10]. Huang et al. (2017) conducted a retrospective cohort study with 56 patients diagnosed with lung and esophageal cancer. Among the patients, there were 45 males and 11 females, with three patients under 50 years old and 53 patients over 50 years old [11]. Iyoda et al. (2021) conducted a retrospective chart review with 106 patients (mean age: 64) diagnosed with central airway stenosis or obstruction due to thoracic malignancy. The study included 80 males and 26 females [12]. Lachkara et al. (2020) conducted a retrospective chart review study on patients with malignant carina involvement treated with bronchial Y stents. The study included 40 patients, with 25 males and 15 females in the Silicone Y stent (SYS) group, and 28 males and 10 females in the SEM Y stent (SEMYS) group [13]. Ma et al. (2008) conducted a retrospective chart review with 72 patients (median age: 57) diagnosed with malignant tumor compression or invasion causing large airway stenosis. The study included 52 males and 20 females [14]. Marchese et al. (2015) conducted a retrospective chart review with 51 lung cancer patients (mean age: 64 ± 11), including 37 males and 14 females [15]. Marchese et al. (2020) conducted a retrospective chart review involving 51 patients with advanced unresectable lung cancer. The mean age was 67, with 41 males and 10 females [16]. Marchioni et al. (2020) conducted a multicentric retrospective study with 100 patients with central airway obstruction, focusing on integrated treatment (60 patients) versus standard treatment (40 patients). The study had a total of 68 male patients and focused on non-small cell lung cancer (NSCLC) at Stage IIIB [17].

In a 2000 prospective multicenter study by Miyazawa et al., 34 patients (30 male, four female) with various cancers, including bronchogenic carcinoma, squamous cell carcinoma, adenocarcinoma, small cell carcinoma, adenoid cystic carcinoma, esophageal carcinoma, mediastinal tumor, and metastatic pulmonary disease, were included. The patients had a mean age of 63.0 years (range: 41-82 years) [18]. In another prospective case-control study by the same authors in 2004, 66 patients (18 female, 48 male) with tracheal, carinal, bronchial, or extensive stenosis were analyzed. The mean ages for different stenosis types were as follows: tracheal stenosis: 61.2 ± 5.9, carinal stenosis: 64.9 ± 5.6, bronchial stenosis: 67.7 ± 5.5, and extensive stenosis: 66.4 ± 3.0 [19]. Monnier et al. conducted a prospective trial in 1996, involving 40 patients (29 male, 11 female) with primary tracheal or bronchial squamous cell carcinoma, esophageal squamous cell carcinoma with a tracheobronchial fistula, and other histologic features. The patients had an average age of 62 years (range: 36-83 years) [20]. Nakajima et al. conducted a retrospective review in 1999, including 22 patients (18 men, four women) with bronchogenic carcinoma, esophageal carcinoma, or thyroid carcinoma. The mean age of the patients was 62 years (range: 52-83 years) [21]. Oki et al. reviewed 30 patients (24 male, four female) with various cancers, including lung cancer, squamous cell carcinoma, adenocarcinoma, small cell carcinoma, esophageal cancer, thyroid cancer, renal cell carcinoma, adenoid cystic carcinoma, malignant lymphoma, tracheal cancer, squamous cell carcinoma, and Ewing sarcoma. The median age of the patients was 62.5 years (IQR: 13-86 years) [22].

In a 2016 retrospective chart review by Özdemir et al., 14 patients (12 male, two female) with non-small cell lung cancer and small cell lung cancer were included [23]. Razi et al. included 50 patients (29 male, 21 female) with various cancers, including non-small cell lung cancer, small cell lung cancer, esophageal cancer, mediastinal sarcoma, and metastatic colon and breast cancer [24]. Righini et al. reviewed 69 patients (40 male, 29 female) with tracheobronchial cancers, esophageal cancer, thyroid cancer, mediastinal malignancy, and other malignancies [25]. Saji et al. included 59 patients (51 male, eight female) with various types of lung cancer [26]. Tayama et al. included 20 patients (eight male, 12 female) with different types of cancer [27]. Verma et al. reviewed 30 patients (22 male, eight female) with malignant airway obstruction [28]. Wilson et al. reviewed 56 patients (33 male, 23 female) with respiratory distress due to malignant obstruction of the trachea and/or a main bronchus [29]. Yerushalmi et al. reviewed 34 patients with malignant airway obstruction, of which 35% had primary lung cancer and 65% had metastatic disease [30]. Zwischenberger et al. included 14 patients (seven male, seven female) with severe dyspnea and different types of non-small cell cancer [31]. Akram et al. reviewed 51 patients (24 male, 27 female) with various types of cancer [32]. Bolliger et al. included 26 patients (16 male, 10 female) with bronchogenic carcinoma, esophageal carcinoma, metastases, tracheal carcinoma, and schwannoma [33]. Chhajed et al. included 93 patients (88 male, 42 female) who underwent stent placement out of a total of 130 patients with lung cancer, esophageal cancer, and pulmonary metastases [34].

Pre-intervention Parameters

Table 3 provides an insight into the pre-intervention parameters.

**Table 3:** Pre-intervention parameters KPS: Karnofsky Performance Status PaO2: Partial pressure of arterial oxygen PaCO2: Partial pressure of arterial carbon di oxide ASA: American Society of Anesthesiologists ECOG: Eastern Cooperative Oncology Group IQR: Interquartile range VC: Vital capacity in liters FVC: Forced vital capacity in liters FEV1: Forced expiratory volume in 1 second in liters PEF: Peak expiratory flow in liters/min PEFR: Peak expiratory flow rate in liters/min MRC: Medical Research Council

	Authors	Pulmonary function testing	Imaging	Site of lesion/location of obstruction	Degree of obstruction	Dyspnea grade/additional scoring systems and scales used	Type of stenosis	Blood gas parameters
1.	Dalar L et al [8].	Not mentioned	Not mentioned	The primary lesions were confined only to the trachea in 65 (11.9%) patients; trachea and right main bronchus in 87 (15.9 %) patients; trachea and left main bronchus in 20 (15.9 %) patients; trachea and both main bronchi in 121 (22.1 %) patients, and the right and left bronchial systems in 9 (1.6%) patients.	Not mentioned	Not mentioned	Not mentioned	Not mentioned
2.	Dutau H et al [9].	Not mentioned	Not mentioned	Not mentioned	Not mentioned	Not mentioned	Not mentioned	Not mentioned
3.	Grosu HB et al [10].	Not mentioned	Not mentioned	Not mentioned	Not mentioned	Not mentioned	Intrinsic compression in 28 patients. Extrinsic compression in 3 patients. Complex in 36 patients. Other/Mixed in 5 patients. 0 patients had fistula formation.	Not mentioned
4.	Huang S et al [11].	Not mentioned	Not mentioned	Not mentioned	Not mentioned	Dyspnea grade: 0 in 13 patients. 1 in 17 patients. 2 in 13 patients. 3 in 3 patients.	Intrinsic compression in 28 patients. Extrinsic compression in 11 patients. Complex obstruction in 0 patients. Fistula formation in 17 patients.	
5.	Iyoda A et al [12].	Not mentioned	Not mentioned	Not mentioned	Not mentioned	Not mentioned	Not mentioned	Not mentioned
6.	Lachkara S et al [13].	Not mentioned	Not mentioned	SYS group: Metastatic disease in 22 patients, locally advance disease in 18 patients. SEMYS group: Metastatic disease in 24 patients, locally advance disease in 14 patients.	Not mentioned	Not mentioned	Not mentioned	Not mentioned
7.	Ma G et al [14].	Not mentioned	Not mentioned	Middle-lower trachea in 45 cases, right main bronchus in 3 cases, left main bronchus in 2 cases, coexisting at the trachea and one-sided bronchus in 2 cases.	Not mentioned	KPS value (Mean ± SD) : 68.58 ± 8.08.	Not mentioned	PaO2 (Mean ± SD): 7.74 ± 0.99. PaC02: (Mean ± SD) 5.37 ± 0.39.
8.	Marchese R et al [15].	Not mentioned	Not mentioned	Not mentioned	Not mentioned	ASA Score (Mean ± SD): 3 ± 0.5. ECOG Score (Mean ± SD): 1.7 ± 0.6. Modified MRC 2.7 ± 0.8	Intrinsic compression in 10 cases. Extrinsic compression in 12 cases. Complex in 27 cases. Fistula in 1 case.	
9.	Marchese R et al [16].	Not mentioned	Not mentioned	The obstructions were noted to be in the left lower lobe bronchus, left upper lobe bronchus, left secondary carina, right lower lobe bronchus, right primary carina and right secondary carina.	Not mentioned	ECOG Score (Mean ± SD): 1.8 ± 0.7. MMRC dyspnea score, (Mean ± SD): 2.6 ± 0.8). Oxygen Saturation (Mean ± SD): 95 % ± 2. Barthel index (Mean ± SD): 82 ± 2.5).	Intrinsic compression in 8 patients. Extrinsic compression in 10 patients. Complex in 31 patient. Fistula formation in 0 patients.	
10.	Marchioni A et al [17].	Not mentioned	Not mentioned	Trachea, n (%): Total, 21 (21). Integrated treatment, 16 (27). Standard treatment, 5 (13). Main right bronchus, n (%): Total, 60 (60). Integrated treatment, 35 (58). Standard treatment, 25 (63). Main left bronchus, n (%): Total, 47 (47). Integrated treatment 29 (48). Standard treatment, 18 (45). Carina, n (%): Total, 17 (17). Integrated treatment, 15 (25). Standard treatment, 2 (5). Extensive involvement, n (%): Total, 17 (17). Integrated treatment, 15 (25). Standard treatment, 2 (5).	Obstruction, % (IQR): Total, 65 (60-75). Integrated treatment 70 (65-75). Standard treatment 65 (65-75).	Not mentioned	Not mentioned	Not mentioned
11.	Miyazawa T et al [18].	VC in liters (Mean ± SD): 1.97 ± 0.54. FVC in liters (Mean ± SD): 1.40 ± 0.51. PEF in liters per second (Mean ± SD): 2.9 ± 1.4.	Not mentioned	Not mentioned	81 ± 15% before stent placement	Dyspnea grade 0, I, II, III, IV in 0, 3, 8, 8, and 15 patients respectively before stent placement.	Intrinsic Compression in 22 cases. Extrinsic Compression in 12 cases.	Not mentioned
12.	Miyazawa T et al [19].	Tracheal stenosis: FVC in liters (Mean ± SD), 2.94 ± 0.95. FEV1 in liters (Mean ± SD), 1.67 ± 0.60. PEF in liters per second (Mean ± SD), 3.14 ± 1.67. Vmax 50% in liters per second (Mean ± SD), 1.70 ± 1.26. Vmax 25% in liters per second (Mean ± SD), 0.82 ± 0.50. Carinal Stenosis: FVC in liters (Mean ± SD), 2.59 ± 0.82. FEV1 in liters (Mean ± SD), 1.56 ± 0.68. PEF in liters per second (Mean ± SD), 2.44 ± 1.22. Vmax 50% in liters per second (Mean ± SD), 1.21 ± 0.80. Vmax 25% in liters per second (Mean ± SD), 0.62 ± 0.65. Bronchial Stenosis: FVC in liters (Mean ± SD), 2.04 ± 0.55. FEV1 in liters (Mean ± SD), 1.46 ± 0.40. PEF in liters per second (Mean ± SD), 3.31 ± 1.47. Vmax 50% in liters per second (Mean ± SD), 1.54 ± 0.70. Vmax 25% in liters per second (Mean ± SD), 0.53 ± 0.31 . Extensive stenosis: FVC in liters (Mean ± SD), 2.22 ± 0.63. FEV1 in liters (Mean ± SD), 1.06 ± 0.36. PEF in liters per second (Mean ± SD), 1.58 ± 0.71. Vmax 50% in liters per second (Mean ± SD), 0.82 ± 0.54. Vmax 25% in liters per second (Mean ± SD), 0.47 ± 0.48.	Not mentioned	Tracheal stenosis in 20 patients, Carinal stenosis in 16 patients, Bronchial stenosis in 18 patients, Extensive stenosis from the trachea, carina, extending to the bronchi due to tumor and/or mediastinal lymphadenopathy in 10 patients.	Not mentioned	Dyspnea grade, number of patients (n): Tracheal stenosis: 0 (0), I (0), II (0), III (9), IV (11). Carinal Stenosis: 0 (0), I (0), II (0), III (9), IV (7). Bronchial stenosis: 0 (0), I (0), II (0), III (13), IV (5). Extensive stenosis: 0 (0), I (0), II (0), III (1), IV (9).	Extrinsic Compression in 64 patients	Not mentioned
13.	Monnier P et al [20].	Not mentioned	Not mentioned	Trachea in 11 patients, at the level of the carina in 6 cases (left -sided prevalence in 4 and right-sided in 2). Tumoral stenosis involved the left main bronchus in 11 cases, the right main or intermediate bronchus in 10 cases, and 1 case each where only the right main or intermediate bronchus was implicated.	The severity of the stenosis ranged from total obstruction to a residual lumen of approximately 6 mm (median diameter, 3.8 mm). Expressed as percentages, the residual lumina represented an average obstruction of 75% (±25%). The length of the stenosis was estimated to be, on average, 34 mm, with extremes ranging from 20 to 50 mm. Bronchial obstruction (degree in %), number of patients (n): 0-25%, 0. 25-50%, 3. 50-75%, 14. 75-90%, 10. 90-100%, 13.	Dyspnea grade 0, 1, 2, 3, and 4 in 1, 2, 7, 17, and 13 patients respectively.	Not mentioned	Not mentioned
14.	Nakajima Y et al [21].	Not mentioned	Not mentioned	The trachea in eight patients, trachea and left main bronchus in nine, trachea and right main bronchus in three, and left main bronchus in two.	Not mentioned	ECOG Score: Four patients were categorized as grade 4, 13 as grade 3 and five as grade 2. Hugh Jones classification: 13 patients were categorized as grade 5 and nine as grade 4.	Intrinsic Compression in 5 patients. Extrinsic Compression in 17 patients.	Not mentioned
15.	Oki M et al [22].	Not mentioned	Not mentioned	Trachea, 16 patients (53%). Trachea and bronchus, 6 patients (20%). Bronchus, 8 patients (26%).	Not mentioned	Not mentioned	Intrinsic Compression, 0 patients. Extrinsic Compression, 7 patients. Complex, 23 patients. Fistula Formation: With atelectasis, 6 patients. Right lung, 2 patients. Left lung, 2 patients. Right middle and lower lobe, 2 patients. No atelectasis, 0 patients.	Not mentioned
16.	Özdemir C et al [23].	Not mentioned	Not mentioned	Not mentioned	Not mentioned	The ASA (Mean ± SD) patient score prior to intervention was 2.64 ± 0.74 (Range 1 to 4).	Not mentioned	Not mentioned
17.	Razi SS et al [24].	Not mentioned	Not mentioned	Not mentioned	Extent of luminal obstruction 75 to 90% in 10 patients (20%), more than 90% in 40 patients (80%).	MRC dyspnea scale score mean value: 4.40. Mean preoperative ASA score was 3.31. Mean preoperative ECOG performance status score of 3.36.	Intrinsic Compression in 5 patients. Extrinsic Compression in 10 patients. Complex in 32 patients. Fistula Formation in 3 patients.	Not mentioned
18.	Righini C et al [25].	Not mentioned	Not mentioned	Tracheal, 43 patients. Tracheobronchial, 11 patients. Bronchial, 15 patients.	Not mentioned	Not mentioned	Not mentioned	Not mentioned
19.	Saji H et al [26].	Not mentioned	Not mentioned	Not mentioned	Not mentioned	Performance status 0-2, 44 patients. Performance status 3-4, 4 patients. Unknown performance status, 11 patients.	Not mentioned	Not mentioned
20.	Tayama K et al [27].	Not mentioned	Not mentioned	The right mainstem bronchus, 1 patient. The left mainstem bronchus in3 patients. The trachea alone in 9 patients. The trachea and one mainstem bronchus in 3 patients. The trachea and both mainstem bronchi in 2 patients.	Not mentioned	Not mentioned	Intrinsic Compression in 15 patients. Extrinsic Compression in 5 patients.	Not mentioned
21.	Verma A et al [28].	Not mentioned	Not mentioned	Tracheal, 10 patients. Left main Bronchus, 16 patients. Right main bronchus, 8 patients. Mixed, 5 patients.	Not mentioned	Not mentioned	Not mentioned	Not mentioned
22.	Wilson GE et al [29].	FEV1 (Mean ± SD), 1.13 ± 0.41. FVC (Mean ± SD), 1.96 ± 0.7. PEFR (Mean ± SD), 134 ± 12.	Not mentioned	Trachea, 20 patients. Right bronchial tree, 10 patients. Left bronchial tree, 12 patients. Both main bronchi, 3 patients. Trachea and left main bronchus, 4 patients. Trachea and right main bronchus, 3 patients. Trachea and both main bronchi, 6 patients.	Thirty patients (53%) had >90% obstruction of a main bronchus and the remaining 26 had partial obstruction (over 50%) of their trachea or of a main bronchus (or both main bronchi) at bronchoscopy examination.	MRC (Mean ± SD) 5 ± 0. Karnofsky score (Mean ± SD) 29.1 ± 11.4. Visual analogue score (Mean ± SD) (breathing), 40 ± 23. Visual analogue score (Mean ± SD) (walking), 51 ± 23.	Not mentioned	PaO2 (Mean ± SD), 8.81 ± 2.7. PaCO2 (Mean ± SD), 5.33 ± 1.0. pH (Mean ± SD), 7.45 ± 0.03.
23.	Yerushalmi R et al [30].	Not mentioned	Not mentioned	Left mainstem bronchus (31%), trachea (26%), right mainstem bronchus (26%), subglottic (14%), and bronchus intermedius (3%).	Not mentioned	Not mentioned	Not mentioned	Not mentioned
24.	Zwischenberger JB et al [31].	Not mentioned	Not mentioned	Not mentioned	Not mentioned	Karnofsky scores in the range of 50 to 70, 9 patients. Greater than 70, 2 patients. Less than 50, 3 patients.	Not mentioned	Not mentioned
25.	Akram MJ et al [32].	Not mentioned	Not mentioned	Bronchial stenosis, 18 patients. Tracheal stenosis, 11 patients. Tracheo-esophageal fistula, 10 patients. Tracheal stenosis and fistula, 7 patients. Bronchial stenosis and fistula, 2 patients. Bronchial fistula, 3 patients.	Not mentioned	ECOG score (Mean ± SD): 3.65 ± 0.6	Intrinsic Compression in 20 patients. Extrinsic Compression in 13 patients Mixed in 5 patients. Fistula formation in 13 patients.	Oxygen Saturation (Mean ± SD): 89.8 ± 6.7. PaO2 (Mean ± SD): 72.3 ± 12.3.
26.	Bolliger CT et al [33].	FEV1 (Mean ± SD) of all 26 patients: 1.2 ± 0.5 FVC (Mean ± SD) of all 26 patient: 2.1 ± 0.7	Not mentioned	Right main bronchus, 10 patients. Left main bronchus, 8 patients. Trachea, 7 patients. Tracheo-bronchial, 2 patients.	Diameter of affected airway <50% of normal after resection of endoluminal components of obstruction, or diameter of >50% of affected airway after resection of endoluminal component of obstruction if no other therapeutic option available (i.e., radio-chemotherapy).	Dyspnea index of all 26 patients (Mean ± SD): 3.3 ± 0.7	Not mentioned	Not mentioned
27.	Chhajed PN et al [34].	FEV1, median (IQR): Out of 87 patients whose spirometry was available: 62% (49 to 72%). FVC, median (IQR): Out of 87 patients whose spirometry was available: 68% (55 to 78%)	Not mentioned	Values given as in number of procedures (total procedures were 167): Trachea, 26. Trachea plus either or both the main bronchi, 20. Left bronchial system, 52. Right bronchial system, 68. Left as well as the right bronchial system, 1.	Airway obstruction more than 50% of the lumen.	Not mentioned	Intrinsic Compression: 101 procedures performed for intrinsic lesions. Extrinsic Compression: 101 procedures performed for extrinsic lesions. Mixed/Combined: 49 procedures performed for combined intrinsic and extrinsic lesions. 2 procedures were performed for treatment of stump insufficiency after pneumonectomy and 5 for treatment of an esophageal tracheobronchial fistula (grouped together as airway insufficiency).	Not mentioned

In a study conducted by Dalar et al., the site of lesion or location of obstruction varied among the patients with airway lesions. The lesions were found to be confined only to the trachea in 65 (11.9%) patients, trachea, and right main bronchus in 87 (15.9%) patients, trachea and left main bronchus in 20 (15.9%) patients, trachea and both main bronchi in 121 (22.1%) patients, and the right and left bronchial systems in nine (1.6%) patients [8]. In the study conducted by Lachkara et al., the details regarding pulmonary function testing or imaging were not provided. The location of the lesion and obstruction was categorized into two groups: SYS group and SEMYS group. The SYS group consisted of 22 patients with metastatic disease and 18 patients with locally advanced disease, while the SEMYS group included 24 patients with metastatic disease and 14 patients with locally advanced disease [13]. Ma et al. also did not mention specific details about pulmonary function testing or imaging. The site of lesion and obstruction involved different areas, including the middle-lower trachea (45 cases), the right main bronchus (three cases), the left main bronchus (two cases), and coexistence of tracheal and one-sided bronchial involvement (two cases). The study reported the Karnofsky Performance Status (KPS) value (68.58 ± 8.08) and blood gas parameters (PaO2: 7.74 ± 0.99, PaCO2: 5.37 ± 0.39) [14]. In the study conducted by Marchese et al., the details about pulmonary function testing or imaging were not mentioned. The study reported the American Society of Anesthesiology (ASA) Score (mean ± SD: 3 ± 0.5), Eastern Cooperative Oncology Group (ECOG) Score (mean ± SD: 1.7 ± 0.6), and Modified Medical Research Council (MMRC) dyspnea score (mean ± SD: 2.7 ± 0.8). The site of lesion and obstruction included intrinsic compression in 10 cases, extrinsic compression in 12 cases, complex involvement in 27 cases, and fistula formation in one case [15].

Similarly, in the study by Marchese et al., no details about pulmonary function testing or imaging were provided. The site of lesion and obstruction involved the left lower lobe bronchus, left upper lobe bronchus, left secondary carina, right lower lobe bronchus, right primary carina, and right secondary carina. The study also reported the ECOG Score (mean ± SD: 1.8 ± 0.7), MMRC dyspnea score (mean ± SD: 2.6 ± 0.8), oxygen saturation (mean ± SD: 95% ± 2), and Barthel index (mean ± SD: 82 ± 2.5). Intrinsic compression was observed in eight patients, extrinsic compression in 10 patients, complex involvement in 31 patients, and no fistula formation was reported. Marchioni et al. did not mention details about pulmonary function testing or imaging in their study. The location of the lesion and obstruction was categorized based on different areas, including the trachea, main right bronchus, main left bronchus, carina, and extensive involvement. The degree of obstruction (IQR) for each category was reported, while no additional information was provided regarding dyspnea grade, stenosis type, or blood gas parameters [16]. Miyazawa et al. reported pulmonary function testing results in their study, including vital capacity (VC), forced vital capacity (FVC), peak expiratory flow (PEF), and various stenosis types. The site of lesion and obstruction included intrinsic compression in 22 cases and extrinsic compression in 12 cases. The study also provided dyspnea grades for patients before stent placement [18]. In another study by Miyazawa et al., pulmonary function testing results were reported for different stenosis types: tracheal stenosis, carinal stenosis, bronchial stenosis, and extensive stenosis. The site of lesion and obstruction involved tracheal stenosis in 20 patients, carinal stenosis in 16 patients [19].

Özdemir et al. did not provide specific information regarding pulmonary function testing, imaging, site of lesion/location of obstruction, or type of stenosis. The degree of obstruction and blood gas parameters were also not mentioned. The ASA patient score prior to intervention was reported to have a mean value of 2.64 ± 0.74, ranging from 1 to 4 [23]. Razi et al. did not mention the specific details of pulmonary function testing, imaging, or site of lesion/location of obstruction. The extent of luminal obstruction was reported, with 10 patients (20%) having an obstruction ranging from 75% to 90% and 40 patients (80%) having an obstruction of more than 90%. The mean value of the MRC dyspnea scale score was 4.40, indicating significant dyspnea. The study also reported the mean preoperative ASA score of 3.31 and the mean preoperative ECOG performance status score of 3.36. The types of stenosis observed in this study included intrinsic compression in five patients, extrinsic compression in 10 patients, complex obstruction in 32 patients, and fistula formation in three patients. The blood gas parameters were not mentioned [24]. Sajia et al. did not provide specific information regarding pulmonary function testing, imaging, or site of lesion/location of obstruction. The degree of obstruction, dyspnea grade, type of stenosis, and blood gas parameters were also not mentioned. However, they reported the performance status of the patients in the study. Out of the total patients, 44 had a performance status of 0-2, indicating good functional ability. Four patients had a performance status of 3-4, indicating limited functional ability. The performance status of 11 patients was unknown. No further information was provided regarding the blood gas parameters or other specific details of the study [26].

The study conducted by Wilson et al. focused on patients with airway obstruction. Pulmonary function testing revealed reduced lung function, with forced expiratory volume in 1 second (FEV1) (mean ± SD) at 1.13 ± 0.41, FVC (mean ± SD) at 1.96 ± 0.7, and peak expiratory flow rate (PEFR) (mean ± SD) at 134 ± 12. The location of obstruction varied, involving the trachea, right and left bronchial trees, and both main bronchi. A significant proportion of patients (53%) experienced severe obstruction (>90%) in a main bronchus, while the remaining patients had partial obstruction in the trachea or a main bronchus. Dyspnea scores indicated significant respiratory impairment, with a mean MRC score of 5 ± 0, KPS of 29.1 ± 11.4, and visual analogue scores for breathing and walking at 40 ± 23 and 51 ± 23, respectively. Blood gas parameters showed compromised oxygenation and ventilation, with a mean PaO2 of 8.81 ± 2.7, PaCO2 of 5.33 ± 1.0, and pH of 7.45 ± 0.03. These findings highlight the severity and impact of airway obstruction on respiratory function in the studied population [29].

One study by Zwischenberger et al. did not provide specific details about pulmonary function testing or imaging. However, Karnofsky scores were reported, with patients scoring between 50 and 70, some scoring above 70, and others scoring below 50 [31]. Akram et al. reported bronchial stenosis, tracheal stenosis, tracheo-esophageal fistula, tracheal stenosis with fistula, bronchial stenosis with fistula, and bronchial fistula as the site of lesion/location of obstruction. They also provided the ECOG score (mean ± SD) of 3.65 ± 0.6. Additionally, they mentioned intrinsic compression, extrinsic compression, mixed compression, and fistula formation in their findings [32]. Bolliger et al. reported the mean FEV1 and FVC values for their 26 patients. The site of lesion/location of obstruction included the right main bronchus, left main bronchus, trachea, and tracheo-bronchial. They also reported a dyspnea index (mean ± SD) of 3.3 ± 0.7 [33]. Chhajed et al. provided spirometry results, including the median FEV1 and FVC values. They reported various locations for procedures, such as the trachea, trachea with one or both main bronchi, left bronchial system, right bronchial system, and both left and right bronchial systems. They also mentioned airway obstruction exceeding 50% of the lumen [34].

Intervention Details

Table 4 provides a summary of the interventional details of endobronchial stent placement.

**Table 4:** Intervention parameters

	Authors	Details of bronchoscope used for stent treatment	Treatment prior to stent therapy	Details of the procedure	Number of treatments/Length of treatment	Adjuvant therapy used alongside stent placement	Post stent therapy
1.	Dalar L et al [8].	Totally, 802 interventional rigid bronchoscopy procedures were applied in 547 patients having a malign airway obstruction.	Not mentioned	The study participants with malignant airway obstruction underwent different therapeutic bronchoscopy interventions such as stent placement, laser, cryotherapy, and Argon plasma coagulation.	Stents were applied during 171 procedures in 147 patients. Overall, 94 Y-stents and 52 tube stents (Novatech, LaCiotat, France) were placed into the central airways of patients having malign airway obstruction. A laser combined with stenting in 36 patients in the present study. Argon plasma coagulation (APC) was combined with stenting in 65 patients in the present study. Cryotherapy was combined with stenting in 5 patients in the present study.	Mechanical debulking: Done as required. Laser therapy (Diode laser therapy): 250 procedures in 178 patients. Cryotherapy: 93 procedures in 54 patients. Argon plasma coagulation: 373 procedures in 257 patients.	For follow up, a flexible bronchoscopy was used in 100 patients after they had stenting.
2.	Dutau H et al [9].	Not mentioned	Forty-three patients were free from any previous oncologic treatment. Of them, 23 patients (group 1) received first-line chemoradiation therapy (14 in the stent arm, 9 in the no stent arm). 20 patients (group 2) received first-line chemotherapy (9 in the stent arm, 11 in the no stent arm) after TB. The remaining 35 patients (group 3) (17 in the stent arm, 18 in the no stent arm) were either considered as failures of first-line oncologic treatment (31 patients) or candidates for palliative care alone (4 patients).	A total of 78 patients (64 males and 14 females) were included over 3 years. After randomization, 40 patients were included in the stent arm and 38 in the no stent arm. Silicone stents were provided by Novatech SA (La Ciotat, France).	A total of 38 patients in the stent arm underwent stent placement and 2 did not receive the allocated stent placement due to intraoperative complications.	Not mentioned	Not mentioned
3.	Grosu HB et al [10].	Not mentioned	Pre-procedure radiation therapy in 38 patients.	Patients were studied who underwent therapeutic bronchoscopy for malignant airway obstruction (including stent placement). For patients with malignant central airway obstruction, stents were placed if (1) there was pure extrinsic compression with > 50% airway occlusion, or (2) if adequate airway patency (> 50%) could not be achieved with ablative techniques alone, or (3) it was felt that airway re-occlusion would occur quickly if a stent was not placed following ablation for a mixed obstruction. Types of stents used: Ultraflex, Aero, Dumon tube stent, Silicone Y-stent and Polyflex.	24 patients underwent stent placement. Ultraflex: a total of 15 stents placed. Aero, a total of 9 stents placed. Dumon tube stent, 1 stent placed. Silicone Y-stent, 3 stents placed. Polyflex, 1 stent placed.	Seventeen of the 24 patients with stents (71%) had ablative therapies concurrent with stent placement.	Chemotherapy: Post procedure chemotherapy in 43 patients. Post procedure radiation therapy in 29 patients.
4.	Huang S et al [11].	The flexible bronchoscopy (BF 1T260, Olympus, Tokyo, Japan)	Not mentioned	Patients with lung and esophageal cancer who underwent stent placement were included in the study. The self-expanding covered metallic stent had a tracheal limb measuring 10 to 22 mm in diameter and 20 to 100 mm in length. For the Y stent, the diameter of the left or right main bronchi varied from 10 to 18 mm, and the length varied from 10 to 40 mm. The size of the stents was customized to fit different patients’ airways. Type of stents: Tube and Y shaped. Stent length: <60mm, 27 stents. >60 mm, 15 stents.	Number of stents: 1, 46 patients. 2, 10 patients. Stents in right main bronchus: 31. Stent in carina: 14. Stent in main trachea: 32. Tube stent, 31 stents placed. Y shaped stent, 25 stents placed	Not mentioned	Not mentioned
5.	Iyoda A et al [12].	Rigid bronchoscopy under general anesthesia for patients undergoing silicone stent placement.	A total of 12 patients underwent chemoradiation prior to stent placement.	Patients with central airway obstruction due to thoracic malignancy were enrolled and underwent either silicone stent (SS) or metallic stent (MS) placement.	SS (number of stents): Dumon, 27. Dumon Y, 18. Ultraflex, 0. Aero, 0. MS (number of stents): Dumon, 0. Dumon Y, 0. Ultraflex 55. Aero, 6.	Not mentioned	14 patients required additional chemo after stent (8 patients who underwent silicone stent and 6 patients who underwent metallic stent placement.
6.	Lachkara S et al [13].	Rigid bronchoscope.	Previous chemo and/or radiotheraprior to stent placement: 9 patients receiving silicone Y stent and 20 patients receiving SEM Y stent placement.	Y shaped stent, silicon based or self-expanding metallic stent placement.	40 patients underwent silicone Y stent placementand 38 patients underwent SEM Y stent placement. 21 auto-expansive esophageal stents were placed after the bronchial stent procedure (9 in the silicone Y group and 12 in the SEM Y group).	Radiation therapy in 12 patients and 12 patients for silicone Y and SEM Y groups, respectively. Mechanical and/or electrocoagulation debulking was performed in19 patients (55.9%) in the silicone Y group and in 19 patients (50%) in the SEM Y group.	After stenting 20 patients (58.8%) in the silicone Y group and 26 patients (68.4%) in the SEM Y group received oncological treatment, including chemotherapy in 18 and 22 patients respectively.
7.	Ma G et al [14].	Bronchoscope (LF-TP-model, Olympus company)	Not mentioned	Stent placement for malignant air way obstruction. All cases were divided into three groups according to the location of the primary tumor: lung cancer group, esophageal carcinoma group and lymphoma group. Three cases with unknown pathologies were not categorized into any groups. Ultraflex self-expandable, non-membrane coated metallic stents were used for all patients.	Stent implantation was performed success- fully in all 52 cases.	Not mentioned	All 33 patients from the lung cancer and the esophageal carcinoma group received postoperative radiotherapy/ chemotherapy, while six patients from the lymphoma group received postoperative chemotherapy.
8.	Marchese R et al [15].	Rigid bronchoscopy (model 1T-180; Olympus America Inc., Melville, N.Y., USA) under local anesthesia.	Not mentioned	Fully covered SEMS Silamet stent placement in malignant airway obstruction.	Stents were implanted in 52 patients. Stents were inserted in the trachea (n = 19), in the main bronchi (n = 21) and in the peripheral bronchi (n = 31).	Laser Therapy: Laser (λ = 980 nm; Ceralas D50/980/600; Biolitech, Bonn, Germany) therapy was used in case of endoluminal lesions.	Three patients needed mechanical ventilation in the postoperative period for less than 8 hours.
9.	Marchese R et al [16].	Stenting procedures were performed using rigid bronchoscope (Dumon-Harrell type; Bryan Corp; Woburn, MA) under general anesthesia and jet ventilation.	Pre-procedural treatment 16 (30%): Chemotherapy 13 (25%) Chemoradiotherapy 3 (5%)	Both metallic and silicone stents were used: fully covered self-expandable metallic stent (SEMS) Silmet® (Novatech, La Ciotat, France); covered Ultraflex® (Boston Scientific, Natick, MA, USA); Dumon stents straight, Y-shape; and Oki stent (Novatech).	A total of 52 stents were placed. Silmet: Linear, 19 Conic, 6. Silicone: Oki, 14 Straight, 5 Y, 3. Ultraflex: 5.	Not mentioned.	Post-procedural treatment: 40 (75%) Chemotherapy 33 (63%). Radiotherapy 2 (3%). Surgery 4 (7%).
10.	Marchioni A et al [17].	A Dumon rigid bronchoscope (Efer Medical, La Ciotat, Cedex, France) under general anesthesia performed in all patients	Traditional chemo and radiotherapy: 55 (92%) patients in the integrated treatment group and 35 in the standard treatment. Immunotherapy: 11 patients (18%) were in the integrated treatment group and 6 (15%) in the standard treatment. Tyrosine kinase inhibitor: 12 patients (12%) in the integrated treatment group and 3 (8%) in the standard treatment.	Patients were divided into 2 groups. 1) integrated treatment-IT (patients undergoing endoscopic treatment plus chemotherapy/radiotherapy); 2) standard treatment-ST (chemotherapy/radiotherapy alone). In cases with extrinsic compression from malignant occlusion, or whenever indicated, a silicone stent (NOVATECH Doumon stents, Boston Medical Products, Inc., Westborough, MA, USA) was placed.	Total number of stenting procedures, 54 (90%). Y shaped stent, 24 (40%). Single, 34 (58%).	Bougies: 16 (46%). Laser + mechanical, 13 (37%). Laser Therapy: 6 (17%).	Not mentioned.
11.	Miyazawa T et al [18].	For stent insertion, a flexible bronchoscope was used in 24 instances, and a rigid bronchoscope was used in 10.	Surgery in 5 patients. Chemotherapy in 8 patients. Radiotherapy in 10 patients.	Implantation of Ultraflex Nitinol stent in malignant airway obstruction.	A total number of 54 Ultraflex stents placed of varying diameter and length.	In 11 patients, debulking was performed using Nd-YAG laser and/or mechanical debulking.	8 patients were subjected to additional radiotherapy/chemotherapy after stent implantation.
12.	Miyazawa T et al [19].	A flexible bronchoscope (BF 240; Olympus) was used to locate the lesion followed by rigid scope (EFER, La Ciotat, France) to place the stent.	Tracheal Stenosis: Chemotherapy, 15 patients. Radiotherapy, 17 patients. Carinal Stenosis: Chemotherapy, 10 patients. Radiotherapy, 11 patients. Bronchial Stenosis: Chemotherapy, 11 patients. Radiotherapy, 14 patients. Extensive Stenosis: Chemotherapy, 9 patients. Radiotherapy, 7 patients.	Dumon stents (Novatech, Aubagne, France) and uncovered Ultraflex stents (Boston Scientific, Natick, MA) placed in patients with tracheobronchial stenosis.	64 Dumon stents including 36 Y stents and 28 uncovered Ultraflex stents were placed.	Not mentioned	Not mentioned.
13.	Monnier P et al [20].	The rigid bronchoscope (Rigid step) was used 23 times and the flexible device (Tele step) was used 27 times.	Most of them had already undergone 1 or more treatments: 15 courses of radiotherapy, 11 pulmona1y resections, 10 palliative laser dilatations, 7 rounds of chemotherapy, and 4 insertions of another stent.	Use of covered Wallstent for the palliative treatment of inoperable tracheobronchial cancers.	50 Wallstents were inserted initially in 40 patients presenting with a tracheal or bronchial tumor.	Except in cases of pure extrinsic compression, the tracheal or bronchial lumen was initially reopened using an Nd-YAG laser.	Additional chemotherapy and/or radiation therapy in 21 patients.
14.	Nakajima Y et al [21].	Flexible bronchoscopy.	Chemotherapy: 9 patients. Radiotherapy: 13 patients. Esophagectomy, 5 patients. Lobectomy, 5 patients. Pneumonectomy, 3 patients.	Placement of Gianturco-Z tracheobronchial stent for malignant airway obstruction. More than one stent was used for complete coverage of the length of the stenosis for tracheal lesions. For lesions involving the tracheal bifurcation and extending into the left main bronchus, a 12-mm bronchial stent was used with a 15-mm tracheal stent partially overlapping the bronchial stent.	A total of 32 stents were placed.	Not mentioned	Patients experiencing stent breakage after 7 weeks analyzed: 3 underwent radiotherapy, 1 underwent bronchial arterial infusion chemotherapy and 3 patients underwent laser treatment.
15.	Oki M et al [22].	Rigid bronchoscopy	Not mentioned.	For airway stenosis, stenting was performed using a silicone straight stent (DUMON; Novatech, La Ciotat, France), silicone bifurcated stent (DUMON or OKI; Novatech), or self-expandable metal stent (Ultraflex, covered type; Boston Scientific, Natick, MA, USA).	Number of stents inserted: Silicone, 23. Straight stent, 2. Bifurcated stent, 12. Two bifurcated stents, 4. Straight stent and bifurcated stent, 3. Straight stent and two bifurcated stents, 2. Metallic, 7.	Bronchoscopic airway reestablishment using argon plasma coagulation, electrocautery, a Cryoprobe, a high-pressure balloon, or the bevel of a rigid bronchoscope prior to stent placement.	Chemo/radiotherapy performed in 21 (70%) patients. Only 3 of 8 patients (38%) who had undergone prior chemoradiotherapy received additional tumor-specific therapy, while 18 of 21 chemoradiotherapy-naïve patients (86%) could receive additional therapy.
16.	Özdemir C et al [23].	All procedures were performed using a rigid bronchoscope (Efer Dumon, EFER Endoscopy, La Ciotat, France) under general anesthesia.	Not mentioned	Self-expandable metallic stent (SEMS) placement for palliation of central airway obstruction.	9 patients received Y shaped SEMS. 2 patients received SEMS of 18 x 14 x 14 mm. 3 patients received SEMS of 20 x 14 x 14 mm.	Mechanical debulking or balloon dilatation: 9 patients. Laser Therapy: 4 patients. Argon plasma coagulation: 2 patients.	Not mentioned
17.	Razi SS et al [24].	Rigid bronchoscope	11 patients underwent chemotherapy and/or radiation prior to airway stenting.	Stents used for malignant airway obstruction were Ultraflex tracheobronchial stent (Bos- ton Scientific), Dynamic (Y) stent systems (Boston Scientific), and AERO stents (Merit Medical Endotek, South Jordan, UT).	Fifty patients received a total of 72 airway stents over a 2-year period, with 65 stents placed at the initial operation. Thirty-eight patients received a single stent, nine received 2 stents, and three patients received 3 stents at the initial operation.	If there was significant endobronchial tumor present, especially if it was felt that a stent could not be satisfactorily deployed, endobronchial tumor resection was performed before stenting (mostly done using bipolar cautery).	During the follow-up period, 10 patients (20%) underwent bronchoscopy evaluation with or without intervention due mucus plugging, stent migration, and for evaluation of disease progression seen on CT scan with planned intervention at the same time. 31 patients underwent chemo and/or radiotherapy after airway stenting.
18.	Righini C et al [25].	Early cases up to 2000 were treated under local anesthesia and using a flexible bronchoscope BF P40 (Olympus Optical, Tokyo, Japan). Subsequently, a rigid ventilating bronchoscope (F7.5 Karl Storz, Tuttingen, Germany) or flexible bronchoscope with intravenous general anesthesia.	Not mentioned	All patients in this study were not suitable for surgical resection and were treated with nitinol stent placement (Ultraflex Microinvasive, Boston Scientific, Watertown, MA).	Total number of stents placed: Trachea, 43. Tracheobronchial, 11. Bronchial, 15.	Balloon dilatation, mechanical debulking, electrosurgery, and laser photo resection as indicated.	Electrosurgery, laser photo resection, and mechanical debulking, were required in 5 patients after complications occurred.
19.	Saji H et al [26].	Rigid and flexible bronchoscopy was performed in almost all patients.	Not mentioned	Airway stenting for advanced lung cancer with central airway obstruction.	Silicon, metallic, or both types of stents were placed in 42 (60%), 19 (29%), or eight (11%) patients respectively. Trachea: 14 stents, 7 metallic and 7 silicon). Carina: 28 stents, 5 dynamic and 23 silicon Y). Right main bronchus, 17 stents, 6 metallic and 11 silicon. Left main bronchus, 17 stents, 15 metallic and 2 silicon). Trunchus intermedius: 4 stents, 2 silicon and 2 metallic. The number of stents required in a patient was single in 53 (83%) patients, double in 10 (14%) patients, and triple in two (3%) patients	Not mentioned	Not mentioned
20.	Tayama K et al [27].	Bronchoscopy was performed under local or intravenous anesthesia to inspect the stricture and clear the airway of any secretions.		Expandable metallic stent placement for central airway obstruction.	The number of stents placed per patient ranged from 1 to 4. A total of 32 stents placed in 20 patients	Laser Therapy: 15 patients with intraluminal obstruction disease. 8 received Nd:YAG laser vaporization before and after stent implement and one patient with extraluminal stenosis due to an adenoid cystic carcinoma received Nd:YAG laser therapy after stent insertion.	Not mentioned
21.	Verma A et al [28].	Rigid bronchoscopy. All other instruments were inserted through the lumen of the rigid bronchoscope.	Routine treatment including chemotherapy and radiation were provided to the patients.	Comparison of Nd:YAG laser therapy versus stent placement for central airway obstruction. 36 patients underwent laser therapy while 30 patients underwent stent placement. Silicone Dumon stents and Ultraflex metallic stents were used.	30 patients underwent stent placement. A total of 39 stents were placed in 30 patients.	2 patients in the stent group required balloon dilatation prior to stent placement. 6 patients underwent both stent and laser treatment.	4 patients in the laser group and 2 patients in the sent group required repeat intervention.
22.	Wilson GE et al [29].	Fiberoptic bronchoscope	Chemotherapy, 4 patients. Radiotherapy, 17 patients. Mechanical debulking: Pneumonectomy, 5 patients. Wedge resection, 1. Sigmoid colectomy, 1. Mastectomy, 2. Esophagogasterectomy, 3.	Use of expandable metal stents for large airway obstruction. Dimensions of stents: 20 mm (width) x 25 mm (length), 20 mm x 50 mm, 30 mm x 25 mm, and 30 mm x 50 mm. In general, the 20 mm width stents were used for obstruction in the main bronchi and 30 mm stents for obstruction in the trachea.	The stents were placed in the tracheo- bronchial tree as follows: Trachea, 19. Left main bronchus 13. Right main bronchus, 10. Both main bronchi, 7. Trachea and left main bronchus, 6. Trachea and right main bronchus, 2. trachea and both main bronchi, 1. The stents overlapped and placed in continuity with each other if the length of tumor was extensive. A total of 117 stents were placed (mean stents 2 per patient, range 1-4).	Not mentioned	Sixteen patients went on to receive radiotherapy and seven received chemotherapy.
23.	Yerushalmi R et al [30].	Olympus 240 video-flexible bronchoscopes (Olympus, Tokyo, Japan).	Not mentioned	Placement of metal Wallstents for malignant airway obstruction	Airway stents were used in 34 patients, including 2 who required 2 stents at different locations, and one who required 2 adjacent stents (total, 37 stents).	If necessary, the endoluminal tumor was resected using Nd:YAG laser or electrocautery. discharge. Eighteen patients (50%) received brachytherapy to the area of obstruction.	During follow-up, recurrent stenoses and obstructions were identified bronchoscopically and were treated by laser resection. All patients underwent at least one bronchoscopy for follow- up and maintenance, except for three who died before such intervention was indicated.
24.	Zwischenberger JB et al [31].	Fiberoptic bronchoscopy.	Radiotherapy, 3 patients.	Metallic stent placement to palliate large airway obstruction in advanced unresectable lung cancer.	Trachea, 1 patient. Main bronchi: Right, 5 patients. Left, 1 patient. Bilateral, 1 patient. Combination trachea and bilateral main bronchi, 1 patient. Combination right main and right upper lobe bronchi, 1 patient. Right upper lobe bronchus, 1 patient.	Not mentioned	Radiotherapy, 4 patients.
25.	Akram MJ et al [32].	Flexible bronchoscopy.	Chemotherapy: All patients. Radiotherapy: 48 patients. Mechanical debulking: 29 patients underwent surgery. Electrocautery done prior to stenting to debulk those lesions which were found not amenable for stenting due to intraluminal and/or extraluminal tumor infiltration.	Fully covered self-expanding metallic stents (FC-SEMS). Variable sized stents were used depending upon scope movement, and distance of lesion from vocal cord and carina.	Left main bronchus stenting, 12 patients. Left lower lobe bronchus stenting, 1 patient. Right main bronchus stenting, 6 patients. Right lower lobe bronchus stenting, 1 patient. Left and right main bronchus stenting, 1 patient. Tracheal stenting, 27 patients. Tracheal and left main bronchus stenting, 3 patients.	11 patients went through both pre and post procedural chemo and radiotherapy.	Not mentioned.
26.	Bolliger CT et al [33].	All procedures were performed by rigid bronchoscopy under general anesthesia.	15 patients had received various cycles of chemo- and/or radiotherapy.	Use of Studded Polyflex stents for neoplastic obstruction of the central airways. The stents used were combinations of various diameters and lengths, with diameters varying from 10 to 18 mm, and lengths from 25 to 60 mm. A total of 27 stents were used.	A total of 27 stents were used. The stents were placed in the following positions: Right main bronchus, 10 stents. Left main bronchus, 8 stents. Trachea, 7 patients. Tracheobronchial, 2 stents.	Laser therapy used for resection of endoluminal components of the central Airway Obstruction. Electrocautery used for resection of endoluminal components of the Central Airway Obstruction.	Bronchoscopy had to be performed 48 ± 24 hours after stent placement. 1 patient with central small-cell lung cancer who under- went radio-chemotherapy, 1 patient with tracheal carcinoma who underwent external beam irradiation.
27.	Chhajed PN et al [34].	Rigid bronchoscopy (Efer-Dumon, Karl Storz Optics; Germany) was performed under general anesthesia in the operating room. Laser ablation (Deka Medical Electronic Associates, Italy) was performed either through the rigid bronchoscope or via the flexible bronchoscope inserted in the rigid bronchoscope.	Not mentioned	Use of stent placement versus laser therapy for malignant airway stenosis. The Dumon stent in the trachea and the right bronchial tree, Ultraflex stent for lesions on the left side. Y stents (Dumon, Polyflex, Dynamic) were used for lesions involving the trachea and both the main bronchi. Overall, laser therapy was used in 127 procedures in 98 patients. Laser therapy as the only therapeutic modality was used during 62 out of 167 (37%) procedures.	108 total stents used. In total, 15 Y-stents and 93 tube stents (Dumon 34, Polyflex 13, Ultraflex 46) were placed. In three patients, two stents were inserted in one procedure. Stents were placed during 105 procedures in 93 patients. Only stent insertion was undertaken in 40 out of 167 (24%) procedures and combined laser followed by stent insertion was performed in 65 out of 167 (39%) procedures.	Not mentioned.	Not mentioned.

In a study by Dalar et al., stents were applied during 171 procedures in 147 patients with malignant airway obstruction, with a total of 94 Y-stents and 52 tube stents placed. Adjuvant therapies such as laser therapy, cryotherapy, and argon plasma coagulation were combined with stenting in various patients. Mechanical debulking, laser therapy, cryotherapy, and argon plasma coagulation were performed as required before stent placement [8]. In another study conducted by Dutau et al., 38 patients underwent stent placement, and the specific type of stents used was not mentioned. The authors investigated patients who received different oncologic treatments before stent therapy [9]. Grosu et al. examined 24 patients who underwent stent placement, with various types of stents used, including Ultraflex, Aero, Dumon tube stent, Silicone Y-stent, and Polyflex. Concurrent ablative therapies were administered to 71% of the patients [10]. Huang et al. included patients with lung and esophageal cancer who underwent stent placement using tube and Y-shaped stents of varying lengths and locations [11]. Iyoda et al. enrolled patients with central airway obstruction due to thoracic malignancy who underwent either silicone stent or metallic stent placement. Additional chemotherapy was required for 14 patients after stent placement (eight patients with silicone stents and six patients with metallic stents) [12].

In the study by Lachkara et al., a total of 78 patients underwent stent placement, with 40 patients receiving Silicone Y stent placement and 38 patients receiving SEM Y stent placement. Post-stent placement, 21 auto-expansive esophageal stents were also placed, with nine in the silicone Y group and 12 in the SEM Y group. Radiation therapy was administered to 12 patients in each group, and mechanical and/or electrocoagulation debulking was performed in 19 patients in the Silicone Y group and 19 patients in the SEM Y group. After stenting, 20 patients in the Silicone Y group and 26 patients in the SEM Y group received oncological treatment, including chemotherapy [13]. In the study conducted by Ma et al., stent placement for malignant airway obstruction was performed using a bronchoscope, and all 52 cases were successfully implanted with Ultraflex self-expandable metallic stents. Among the patients, 33 from the lung cancer and esophageal carcinoma group received postoperative radiotherapy/chemotherapy, while six patients from the lymphoma group received postoperative chemotherapy [14]. Marchese et al. reported the use of fully covered SEMS Silamet stents in 52 patients with malignant airway obstruction, inserted in the trachea, main bronchi, and peripheral bronchi. Laser therapy was employed for endoluminal lesions. In the postoperative period, three patients required mechanical ventilation for less than eight hours [15]. In another study by Marchese et al., stenting procedures were performed using a rigid bronchoscope under general anesthesia and jet ventilation. Chemotherapy was administered in 13 patients, while three patients received chemoradiotherapy. Both metallic and silicone stents were used, with a total of 52 stents placed. Post-procedural treatment included chemotherapy in 33 patients, radiotherapy in two patients, and surgery in four patients [16]. Marchioni et al. conducted a study where stenting procedures were performed using a Dumon rigid bronchoscope under general anesthesia. Patients were divided into two groups: integrated treatment (endoscopic treatment plus chemotherapy/radiotherapy) and standard treatment (chemotherapy/radiotherapy alone). A total of 54 stenting procedures were performed, with Y-shaped stents used in 24 cases and single stents in 34 cases. Various treatment modalities such as bougies, laser therapy, and mechanical debulking were employed, but specific post-procedural treatments were not mentioned [17].

Miyazawa et al. utilized both flexible and rigid bronchoscopes for stent insertion, with a total of 54 Ultraflex Nitinol stents placed in cases of malignant airway obstruction. Debunking procedures were performed in 11 patients using Nd-YAG laser and/or mechanical debulking, and additional radiotherapy/chemotherapy was administered to eight patients after stent implantation [18]. In another study by Miyazawa et al., tracheal, carinal, bronchial, and extensive stenosis cases were treated with Dumon stents and uncovered Ultraflex stents, with a total of 64 stents placed [19]. Monnier et al. employed the use of covered Wallstents for palliative treatment in 40 patients with inoperable tracheobronchial cancers, with additional chemotherapy and/or radiation therapy given to 21 patients [20]. Nakajima et al. performed stent placement using Gianturco-Z tracheobronchial stents in patients with malignant airway obstruction, with a total of 32 stents placed [21]. Oki et al. used various types of stents, including silicone straight stents, silicone bifurcated stents, and self-expandable metal stents, with a total of 23 silicone stents and seven metallic stents inserted [22]. Özdemir et al. utilized self-expandable metallic stents (SEMS) for palliation of central airway obstruction, with nine patients receiving Y-shaped SEMS and others receiving different sizes of SEMS [23]. Razi et al. placed a total of 72 airway stents, including Ultraflex tracheobronchial stents, Dynamic stent systems, and AERO stents, in 50 patients, and some patients underwent chemotherapy and/or radiation prior to stenting [24].

In a study conducted by Righini et al., early cases up to 2000 were treated using a flexible bronchoscope under local anesthesia. Subsequently, a rigid ventilating bronchoscope or a flexible bronchoscope with intravenous general anesthesia was used. All patients in this study, who were not suitable for surgical resection, underwent nitinol stent placement. Additional procedures such as balloon dilatation, mechanical debulking, electrosurgery, and laser photo resection were performed as indicated, with five patients requiring these procedures after complications occurred [25]. Another study by Sajia et al. utilized both rigid and flexible bronchoscopy in the treatment of central airway obstruction in advanced lung cancer. The study employed silicon, metallic, or both types of stents in different patients. Stent placement occurred in various locations, including the trachea, carina, right main bronchus, left main bronchus, and trunchus intermedius. Most patients received a single stent, while a smaller number required double or triple stents [26]. Tayama et al. conducted a study focusing on the placement of expandable metallic stents for central airway obstruction. Bronchoscopy was performed under local or intravenous anesthesia to inspect strictures and clear the airway of any secretions. Laser therapy was used in patients with intraluminal obstruction disease, and one patient with extraluminal stenosis received Nd:YAG laser therapy after stent insertion [27].

In a comparative study by Verma et al., Nd:YAG laser therapy was compared with stent placement for central airway obstruction. Stent placement was performed using Silicone Dumon stents and Ultraflex metallic stents, with some patients requiring balloon dilatation prior to stent placement. Repeat intervention was required for a subset of patients in both groups [28]. Wilson et al. conducted a study focusing on the use of expandable metal stents for large airway obstruction. Different dimensions of stents were used based on the location of the obstruction, with an average of two stents placed per patient [29]. Yerushalmi et al. utilized an Olympus 240 video-flexible bronchoscope for the placement of metal Wallstents in patients with malignant airway obstruction. Follow-up bronchoscopies were performed, and recurrent stenoses and obstructions were treated by laser resection [30]. Zwischenberger et al. used fiberoptic bronchoscopy for the placement of metallic stents to palliate large airway obstruction in advanced unresectable lung cancer. Radiotherapy was administered to some patients as an additional treatment modality [31]. Akram et al. employed flexible bronchoscopy for various treatments such as chemotherapy, radiotherapy, and mechanical debulking. Self-expanding metallic stents were used for airway stenting, and some patients received both pre- and post-procedural chemotherapy and radiotherapy [32].

Bolliger et al. utilized rigid bronchoscopy under general anesthesia for the placement of studded Polyflex stents in neoplastic obstruction of the central airways. Follow-up bronchoscopies were performed within a specific timeframe after stent placement [33]. Chhajed et al. compared stent placement versus laser therapy for malignant airway stenosis. Different types of stents were used depending on the location of the lesion, and laser therapy was used in most procedures [34].

Outcomes and Complications

Table 5 gives a summary of the outcomes and complications of endobronchial valve placement.

**Table 5:** Outcomes and Complications KPS: Karnofsky score MRC: Medical Research Council FVC: Forced expiratory capacity in liters FEV1: Forced expiratory volume in 1 second in liters PEF: Peak expiratory flow in liters per minute PEFR: Peak expiratory flow rate in liters per minute ECOG: European Cooperative Oncology Group SE: Standard error SD: Standard deviation

	Authors	Post procedure stenosis	Post procedure pulmonary function testing	Post procedure dyspnea grade/additional scoring systems and scales used	Post procedure blood gas parameters	Survival outcomes	Other	Complications, n (number of patients)
1.	Dalar L et al [8].	Not mentioned	Not mentioned	Not mentioned	Not mentioned	Median follow-up period: 5.3 months (range 0–100 months). Factors significantly affecting survival: Type of malignancy causing central airway obstruction (p<0.01). Site of lesion (p< 0.01) The type of endobronchial treatment modality (p=0.01). Survival (mean months with 95% confidence interval, % at 3 and 6 months): Laser only: 22.4 (16-27.8), 71 and 57. Argon plasma coagulation only: 29.7 (21.7-37.9), 58 and 49. Cryotherapy only: 20.9 (3.9-37.8), 57 and 35. Stent only: 10.7 (5.9-15.4), 38 and 26. Laser and stent: 7.9 (1.8-14.1), 42 and 11. Argon plasma coagulation and stent: 11.9 (5.7-18.1), 48 and 38.		Complication Rate: 10.8% (59 out of 547). Stent obstruction due to tumor overgrowth: 20. Complications following endobronchial treatment: 59. Arrhythmias during endobronchial treatment: 4. Hypertensive attack: 9 Oxygen desaturation: 24. Restenosis due to the tumor progression: 20.
2.	Dutau H et al [9].	Not mentioned	Not mentioned	Not mentioned	Not mentioned	At the end of the follow up period: 11 out of 40 patients were alive in the stent arm. 10 out of 38 patients were alive in the no stent arm (7 had no recurrence, 3 had recurrence and 1 patient required re-stenting). Survival not affected by stent placement, non-significant improvements in survival times). Survival appeared to be affected by local recurrence.	Not mentioned	Death causes: Progressive cachexia: Stent arm: 17 (42.5%). No stent arm: 17 (44.7%). Progressive bronchial obstruction: Stent arm: 2 (5%). No stent arm: 3 (7.9%). Metastases: Stent arm: 2 (5%). No stent arm: 3 (7.9%). Other (including hemoptysis): Stent arm: 7 (17.5%). No stent arm: 5 (1.1%). Unknown: Stent arm: 1 (2.5%). No stent arm: 0 (0%).
3.	Grosu HB et al [10].	Degree of post procedure airway obstruction: 0 to 49%, 21 patients. 50-100%, 6 patients.	Not mentioned	Not mentioned	Not mentioned	Not mentioned	Not mentioned	Lower respiratory tract infection: 23 (Acute bronchitis: 5. Pneumonia, not in obstruction/stent area: 2. Pneumonia distal to obstruction/stent area: 10. Pneumonia, multi-lobar: 6. Mortality/death: 32. Granulation tissue: 3. Stent obstruction due to tumor overgrowth: 15. Interventions performed for restenosis: 13. New stent placed for restenosis: 5. Stent removal required: 1. Migration: 1 Retained secretions/mucoid impaction: 5. Stent fracture: 3.
4.	Huang S et al [11].	Not mentioned	Not mentioned	The 24-hour post-stent placement mean KPS (Mean ± SD) significantly improved (79.05 ± 20.71 vs. 56.67 ± 23.52, P<0.001). By the subgroup analysis, the KPS improved in both the Lung cancer and Esophageal cancer groups (Lung cancer group: 55.45 ± 21.15 vs. 75.45 ± 22.07; P=0.001, and Esophageal cancer group: 54.29 ± 24.72 vs. 83.57 ± 16.92; P<0.001).	Not mentioned	Not mentioned	Follow up: 545 days	Infection: 3. Granulation tissue: 7. Stent obstruction due to tumor overgrowth: 3. Migration: 5. Retained secretions/mucoid impaction: 7. Stent malposition: 1. Bleeding: 1. Vocal cord paralysis: 4. Fistula: 2. Atelectasis: 1 Double placement: 10.
5.	Iyoda A et al [12].	Not mentioned	Not mentioned	Not mentioned	Not mentioned	After stenting, Median survival times: SS: 5.585 months, MS: 3.220 months. 1-year survival rates: were SS: 25.1% MS: 5.1%. 2-year survival rates: SS: 15.7% MS: 5.1%, SS patients had significantly better prognoses than MS patients (p = 0.0173).	Not mentioned	Infections: 1 (MS 1). 30-day mortality: SS 6, MS 11. On the 30-day mortality rate, there were no significant differences between SS and MS. Granulation tissue: SS 2, MS 3. Stent obstruction due to tumor overgrowth: SS 1. Migration: SS 5, MS 3. Retained secretions/mucoid impaction: SS 4, MS 6. Bleeding: SS 2. Halitosis: MS 1.
6.	Lachkara S et al [13].	Not mentioned	Not mentioned	Not mentioned	Not mentioned	Mortality/death: Silicone Y group, 32 (80%) patients SEM Y group, 34 (89%) patients. Median survival: Silicone Y group, 171 days (IQR 53-379 days) SEM Y group, 104 days (IQR 53-230).	Symptom relief: Silicone Y group, 27 patients SEM Y group, 32 patients. Mean duration of stent in days: Silicone Y group, 150.2 days SEM Y group, 112 days. Stent removal: Silicone Y group, 9 days SEM Y group, 7 days.	Early complications (less than 7 days): Silicone Y group, 9 (27%) SEM Y group, 6 (15%). Late complications (more than 7 days): Silicone Y group, 15 (46%) SEM Y group, 23 (59%). Complications: Silicone Y group, 18 (55%) SEM Y group, 25 (65%).
7.	Ma G et al [14].	Not mentioned	Not mentioned	KPS value: 84.62 ± 5.03.	PaO2: 11.12 ± 0.61. PaCO2: 4.58 ± 0.30.	Three-year survival rates: Lung cancer group, 10 % Esophageal carcinoma group, 7.7% Lymphoma group, 66.7%. Average survival period: Lung cancer group, 16.3 Esophageal cancer group, 9.07 Lymphoma group 35.5 months. The three-year survival rate was significantly higher in lymphoma group than in lung cancer or esophageal cancer group (p < 0.01).	Not mentioned	Infections: 4. Stent obstruction due to tumor overgrowth: 8. Chest pain: 25. Mild fever: 5.
8.	Marchese R et al [15].	Not mentioned	Not mentioned	Statistically significant score difference in the Barthel Index: Median 69 (range 25-93) immediately post procedure, Median 90, (range 35-100) 24 hours after the procedure; p < 0.001. MRC score: Median 3 immediately post procedure, Median 1 24 hours after the procedure; p < 0.001.	Not mentioned	Follow-up, days Mean 119±120 Range 22–549 Median 74.	A radiographic improvement was detected in 48% of patients.	Infections: 3 (5.7%). Granulation tissue in 2 (3.8%). Stent obstruction due to tumor overgrowth in 8 (15%). Stent migration in 7 (13.4%): Post chemotherapy regression of tumor 3 (5.7%) Intraprocedural dislocation 1 (1.9%) Dislocation into cavity abscess 1 (1.9%) Stent-related migration in 2 (3.8). Three patients needed mechanical ventilation in the postoperative period for less than 8 hours, and 2 experienced atrial fibrillation treated with pharmacological cardioversion with success.
9.	Marchese R et al [16].	Not mentioned	Not mentioned	MMRC (modified Medical Research Counsel) dyspnea score (Mean ± SD) (2.6 ± 0.8 vs 1.2 ± 0.5; p <0.01) (immediate post procedure period and 1 month). Oxygen saturation (Mean ± SD) (95 ± 2 vs 96 ± 2.4; p <0.01) (immediate post procedure period and 1 month).	Not mentioned	Discharge occurred 2 ± 3 days after the procedure and the mean follow-up duration was 123 days ± 157 (range: 15–653 days). The median overall survival was 118 ± 21days. The survival of patients with a double airway stent was worse than patients with a single one (p <0.01).	Not mentioned	Early complications after bronchoscopy intervention: Atrial fibrillation 2 (3.9%). Respiratory distress (non-invasive ventilation) 3 (5.8%). Pneumonia 1 (1.9%) Obstruction due to tenacious secretions 1 (1.9%). Infections: 7 (13%). Granulation tissue in 4 (7.6%). Stent obstruction due to tumor overgrowth in 5 (9.8%). Migration: Post-chemotherapy regression of tumor 2 (3.9%). Dislocation into cavitary abscess 2 (3.9%). Stent related migration 2 (3.9%).
10.	Marchioni A et al [17].	Not mentioned	Not mentioned	Not mentioned	Not mentioned	Median follow-up from diagnosis was 21 (IQR 9-36) months. Overall survival was longer in IT (Interventional Bronchoscopy AND chemo-radio) group vs ST (standard Treatment and chemo-radio) although not statistically significant (23.7 months vs 19.2 months, p = 0.2). IT group showed a significantly higher survival gain over ST when patients had KRAS mutation (7.6 months vs 0.8 months, p <0.0001), a lumen occlusion>65% (6.6 months vs 2.9 months, p <0.001), and no involvement of left bronchus (7 months vs 2.3 months, <0.0001). Finally, IT showed a statistically significant favorable difference in terms of overall new hospitalizations (p = 0.03), symptom free interval (p = 0.02), and onset of atelectasis (p = 0.01), but not for occurrence of infections or hemorrhage (p = 0.7 and p = 0.8 respectively, onset of respiratory failure (p = 0.1), use of palliative care (p = 0.9).	Not mentioned	Granulation tissue (8). Complications at 1-year 10 (19): Post-obstructive pneumonia, n (%) 5 (9). Granulation, n (%) 8 (15). Dislocation, n (%) 8 (15). Removal, n (%) 9 (17). Occlusion, n (%) 6 (20).
11.	Miyazawa T et al [18].	Significant improvement in obstruction of airway diameter (Mean % ± SD) : 81.6 ± 15% before vs 14.6 ± 17% on day 1, 12.6 ± 12% on day 30, and 22.6 ± 28% on day 60; p, 0.001.	VC in liters: 2.46 ± 0.60 (p <0.01). FEV1 in liters: 1.74 ± 0.52 (p <0.001). PEF in liters/second: 3.6 ± 1.2 (p<0.05). The flow volume loop after implantation of the stent showed immediate improvement of flow limitation.	The dyspnea index improved significantly after implantation (before vs days 1, 30, and 60; p, 0.001).	Not mentioned	The median survival time of patients was 3 months. The 1-year survival rate was 25.4%	Symptom improvement immediately post procedure in 82% of the patients.	Mortality: 15 cases of cachexia, 5 cases of bleeding, and 1 case of respiratory insufficiency. Granulation Tissue: 1. Stent obstruction due to tumor overgrowth: 2-month follow-up period were tumor ingrowth (24%) and tumor overgrowth (21%). Retained secretions/mucoid impaction in 9.
12.	Miyazawa T et al [19].	Not mentioned	Statistically significant improvement in spirometry and flow volume curves in all stenoses (values mentioned as mean ± SD). Tracheal stenosis: FVC = 3.15 ± 1.87. FEV1 = 2.32 ± 0.57. PEF = 4.69 ± 1.36. Vmax 50% = 2.42 ± 1.20. Vmax 25% = 0.87 ± 0.70. Carinal Stenosis: FVC= 2.73 ± 0.66. FEV1= 2.04 ± 0.55. PEF = 4.57 ± 1.74. Vmax 50% = 2.01 ± 1.00 Vmax25% = 0.59 ± 0.35. Bronchial Stenosis: FVC = 2.50 ± 0.79. FEV1 = 1.79 ± 0.55. PEF = 3.92 ± 1.82. Vmax 50% = 1.65 ± 0.72. Vmax 25% = 0.62 ± 0.35. Extensive stenosis: FVC = 2.33 ± 0.63 (After 1^st^ stenting), 2.70 ± 0.53 (After 2^nd^ stenting). FEV1 = 1.33 ± 0.50 (After 1^st^ stenting), 1.91 ± 0.41 (After 2^nd^ stenting). PEF = 2.53 ± 1.35 (After 1^st^ stenting), 3.89 ± 0.91 (After 2^nd^ stenting). Vmax 50% = 0.94 ± 0.42 (After 1^st^ stenting), 1.99 ± 0.83 (After 2^nd^ stenting). Vmax 25% = 0.34 ± 0.29 (After 1^st^ stenting), 0.58 ± 0.42 (After 2^nd^ stenting).	The dyspnea grades (World Health Organization Index) improved significantly in tracheal, carinal, bronchial, and extensive stenosis groups. Tracheal stenosis: 0 (6), I (14), II (0), III (0), IV (0). Carinal Stenosis: 0 (8), I (8), II (0), III (0), IV (0). Bronchial stenosis: 0 (8), I (10), II (0), III (0), IV (0). Extensive stenosis: After 1^st^ stenting: 0 (0), I (0), II (3), III (7), IV (0). After 2^nd^ stenting: 0 (3) ,I (7), II (0), III (0), IV (0).	Not mentioned	Median survival times (in months ± SD ) after stenting in the groups were as follows: Tracheal stenosis group, 5.9 ± 5.0; carinal stenosis group, 5.6 ± 2.6; bronchial stenosis group, 5.5 ± 3.0; and extensive stenosis group, 3.0 ± 1.0 months.	Not mentioned	Granulation tissue in 22%. Stent obstruction due to tumor overgrowth in 28%. Migration in 8%. Retained secretions/mucoid impaction in 31%.
13.	Monnier P et al [20].	Bronchial obstruction (degree in %) post operative, on day 30 and on day 90/number of patients: 0-25%: 39, 10, 7. 25-50%: 0, 2, 0. 50-75%: 0, 5, 2. 75-90%: 0, 2, 1. 90-100%: 0, 0, 0.	Not mentioned	The average Kamofsky Performance Index improved from 40 to 70 after prosthesis deployment. Dyspnea grade evaluation on day 1, 30 and 90/number of patients: 0: 7, 2: 2. 1: 14, 5: 4. 2: 13, 11: 5, 3: 3, 2: 1, 4: 2, 2: 1.	Not mentioned	Not mentioned	Not mentioned	Stent migration: 5. Retained secretions: 15. Granulation Tissue: 4. Stent obstruction due to tumor overgrowth: 10. No serious complications (death, perforation, hemorrhage, inability to remove an improperly placed prosthesis).
14.	Nakajima Y et al [21].	Not mentioned	Not mentioned	Hugh-Jones score improved by at least 1 point in 21 patients (95%). ECOG performance scale improved by al least 1 point in 17 patients (77%).	Not mentioned	Survival after stent placement was from 2 to 32 weeks with a mean of 15 weeks.	Technical success rate: In all the 22 patients (100%).	Migration: 4 presenting lethal hemoptysis. Retained secretions/mucoid impaction: 4. Stent fracture: 2
15	Oki M et al [22].	Not mentioned	Not mentioned	Not mentioned	Not mentioned	The survival time was significantly longer in patients who received chemotherapy and/or radiation therapy after the procedure (234 vs 37 days, respectively; p <0.001) and in chemoradiotherapy-naive patients (234 vs. 40 days, respectively; p <0.001). The median survival duration after stenting at the time of analysis was 198 days (range, 13–3,009 days).	Not mentioned	Extubation within 48 hours after stenting could be performed in 28 of 30 patients (93%). 2 patients underwent tracheostomy due to retained secretions. Granulation tissue formation in 1 patient. Pneumonia/infection in 1 patient. Additional chemo and/or radiotherapy in 21 patients (70%).
16	Özdemir C et al [23].	Not mentioned	Not mentioned	Not mentioned	Not mentioned	Not mentioned	Not mentioned	Respiratory insufficiency requiring noninvasive ventilation: 2. Minimal to moderate hemorrhage: 6. Retained secretions/mucoid impaction: 3.
17.	Razi SS et al [24].	Not mentioned	Not mentioned	The mean preoperative MRC dyspnea scale score of 4.40 significantly improved to 3.29 postoperatively. Mean preoperative ECOG performance status score of 3.36 significantly improved to 2.32 postoperatively.	Not mentioned	Improved survival (p 0.05) in patients with intermediate performance status, with a median survival of approximately 8 months. A significantly lower survival rate (3-month median survival) was observed in patients with a high preoperative MRC dyspnea score of 5 (hazard ratio 0.40, 95% confidence interval 0.19-0.84) as well as in patients with high preoperative ECOG performance status score of 4 (hazard ratio 0.33, 95% confidence interval 0.15 to 0.70). The overall mean survival was 128 ±15 days, with a median survival of 117 days. The overall 3-month and 6- month survival was 60% and 40%, respectively.	Performance status improved in 45 patients (90%). Significant improvement in performance status was observed in both poor and intermediate performance groups (p, 0.05).	During the follow-up period, 10 patients (20%) underwent bronchoscopy evaluation with or without intervention due to the following reasons: Mucus plugging, stent migration, and for evaluation of disease progression seen on computed tomographic scan with planned intervention at the same time.
18.	Righini C et al [25].	Not mentioned	Not mentioned	Not mentioned	Not mentioned	Median survival time of these patients was 3.7 months. Duration of follow-up ranged between 1 and 1067 days, with a median of 35 days.	There was a decrease in the level of respiratory support after stent placement but this was not significant (p = 0.06). Breathing room air/number of patients: 48 before and 53 after stent placement. Oxygen therapy by nasal canula or mask/number of patients: 18 before and 15 after stent placement. Noninvasive ventilation/number of patients: 1 before and 1 after stent placement. Invasive ventilation/number of patients: 1 before and 0 after stent placement.	Infection: 2. Granulation tissue: 1. Migration: 2. Stent fracture: 2.
19.	Saji H et al [26].	Not mentioned	Not mentioned	Not mentioned	Not mentioned	25.2% of one-year survival rate and 6.2 months of median survival time.	Not mentioned	Mortality: 5. Pneumothorax: 3. Severe mucus: 6. Idiopathic pneumothorax: 3. Idiopathic pyothorax: 2. Esophageal stenosis: 2. Acute pulmonary distress: 2.
20.	Tayama K et al [27].	Not mentioned	Not mentioned	Not mentioned	Not mentioned	The mean follow-up was 2.5 years (with follow-up ranging from 140 to 1610 days).	The patients with extraluminal compression all exhibited marked improvement in their respiratory symptoms after stenting. The patients with an intraluminal obstruction in whom the tumor reduced the lumen by < 50% of the endoluminal diameter also benefited from stenting. In the patients with an intraluminal obstruction or in whom the tumor reduced the lumen by > 50% of the endoluminal diameter, only a slight improvement was observed after stenting.	Stent obstruction due to tumor overgrowth: 8. Bleeding: 9.
21.	Verma A et al [28].	Not mentioned	Not mentioned	Not mentioned	Not mentioned	Number of deaths: Laser only: 21 (58.3%). UFS only: 25 (83.3%). Both Laser and UFS: 5 (83.3%). Survival (months), median (range): Laser only: 12.4 (0.32-76.4) UFS only: 4.6 (0.32-52.1). Both Laser and UFS: 5.9 (0.99-56.2). Survival in trachea and main bronchi group (months), median (range): Laser only: 12 (0.32-76.4) UFS only: 4.6 (0.32-52.1). Both Laser and UFS: 5.9 (0.99-56.2). Survival in lobar bronchi group (months), median (range): Laser only: 45.6 (4.8-67.2).. Survival in patients requiring single rigid bronchoscopy (months), median (range): Laser only: 11.2 (0.32-76.4) UFS only 3.9 (0.32-52.1). Both Laser and UFS: 4.8 (0.99-8.8). Survival in patients requiring multiple rigid Bronchoscopy (months), median (range): Laser only: 27.5 (4.2-64.5) UFS only: 6.3 (0.52-19.5). Both Laser and UFS: 56.3. Survival in patients with definitive treatment (months), median (range): Laser only: 12 (4.2-62.1) UFS only: 9.9 (1.2-42.3). Both Laser and UFS: 8 (0.99-56.3). 30-day mortality/number of patients: Laser only: 3 (8.3%) UFS only: 4 (13.3%) Both Laser and UFS: 1 (16.6%). 1 year mortality/number of patients: Laser only: 14 (38.9%) UFS only: 21 (70%) Both Laser and UFS: 5 (83.4%).	Not mentioned	Any complication: Laser only: 7 (19.4%).. UFS only: 7 (23.3%). Both Laser and UFS: 0 (0%). Escalation of level of care: Laser only: 6 (16.6%). UFS only: 6 (20%). Both Laser and UFS: 0 (0%). Escalation of level of care to ICU: Laser only: 4 (11.1%). UFS only: 1 (3.3%). Both Laser and UFS: 0 (0%). 30-day mortality: Laser only: 3 (8.3%). UFS only: 4 (13.3%). Both Laser and UFS: 0 (0%). Significant bleeding: Laser only: 2 (5.5%). UFS only: 0 (0%). Both Laser and UFS: 0 (0%). Unexpected respiratory failure in 24 hours: Laser only: 1 (2.7%). UFS only: 1 (3.3%). Both Laser and UFS: 0 (0%). Complication requiring CPR: Laser only: 2 (5.5%). UFS only: 0 (0%). Both Laser and UFS: 0 (0%).
22.	Wilson GE et al [29].	Not mentioned	FEV1 in liters (Mean ± SD) = 1.38 (0.57) (p, 0.001). FVC in liters (Mean ± SD) = 2.15 (0.76) (p, <0.05). PEF rate (liter/min) (mean ± SD) = 158 (14) (p, <0.05).	MRC (Mean ± SD) = 4 ± 1. Karnofsky (Mean ± SD) = 51.8 ± 21.4. Visual analogue score, breathing (Mean ± SD) = 63 ± 22. Visual analogue score, walking (Mean ± SD) = 65 ± 25.	pH (Mean ± SD) = 7.37 ± 0.43 (results non-significant). PaO2 (Mean ± SD) = 10.24 ± 3.14 (p, <0.05). PaCO2 (Mean ± SD) = 5.4 ± 1.2 (results non-significant).	The median length of hospital stay was five days (range 1-24 days) Of the 56 patients stented, five were alive after a mean of 207 days (range 135-274) and 51 died with a mean survival of 77 days (range 1-477).	Not mentioned	Infections: 1. Mortality: 4.
23.	Yerushalmi R et al [30].	Not mentioned	Six patients (18%) had pulmonary function both before and after stent insertion, and all showed an improvement in forced expiratory volume in 1 second (5-35%) and forced vital capacity (5-15%).	Degree of dyspnea Improved.	Not mentioned	Median Survival: 6 months (range 0.25-105 months).	Not mentioned	Stent obstruction due to tumor overgrowth: 3.
24.	Zwischenberger JB et al [31].	All patients had successful stent deployment with initial relief of airway stenosis (>75% predicted diameter).	Not mentioned	Stent placement improved the dyspnea score in 7. patients (50%), with symptoms unchanged in the remainder. Upon follow up questioning, at the time of perceived maximum benefit, 6 patients felt they had achieved significant improvement in functional status. Of the 9 patients surviving greater than 2 months, the Karnofsky score improved in 4 and was unchanged in 5.	Not mentioned	Total length of stay ranged from 3 to 22 days (average, 10.2 days). 10 of the patients were deceased at 8-month follow up. Five patients died in less than 2 months. All 4 patients with stage IV disease died within 2 months of the procedure. Of those who died, 5 were able to return home before death, 4 never left the hospital or required early readmission, and 1 died at a nursing care facility.	Not mentioned	Problems identified in individuals before death included a tracheoesophageal fistula, ipsilateral pneumothorax, tracheostomy, and atrial fibrillation.
25.	Akram MJ et al [32].	Not mentioned	Not mentioned	Not mentioned	Partial pressure arterial oxygen: Mean difference ± SE: -18.16 ± 2.50. There was a statistically significant mean difference in pre- and post-procedure partial pressure arterial oxygen (Mean = 72.3, SD = 12.3 vs Mean = 90.5, SD = 15.1, p = 0.001).	The overall median (SD) survival time was 16 (3.44). The median (SD) survival time was highest in intrinsic compression of the airway [27.00 (6.51) weeks] compared to that in extrinsic compression and trachea-esophageal fistula [16.00 (9.12) and 8.00 (2.34)] weeks, respectively. Patients who received pre- and post-procedure chemotherapy and radiotherapy had a better median (SD) survival [28.00 (12.11) versus 11.00 (2.15)], p-value <0.04.	Oxygen saturation: Mean difference ± SE: -5.72 ± 0.99. There was a statistically significant mean difference in pre- and post-procedure oxygen saturation (Mean = 89.8, SD = 6.7 vs Mean = 95.5, SD = 2.54, p = 0.001). White blood cell count: Mean difference ± SE: 0.86 ± 0.63. Performance status: Mean difference ± SE: 1.06 ± 0.10. There was a statistically significant difference in pre- and post-procedure performance status (Mean = 3.65, SD = 0.6 vs Mean = 2.59, SD = 0.83, p = 0.001). Serum albumin: Mean difference ± SE: -0.35 ± 0.11. Hemoglobin: Mean difference ± SE: -0.45 ± 0.20. 29 (56.9%) patients had symptomatic improvement.	No complications: 29 (56.9). Acute Pneumothorax: 1 (2.0%). Mucous plugging: 2 (3.9%). Stent obstruction: 4 (7.8%). Recurrent Pneumonia: 5 (9.8). Stent migration: 8 (15.7). Acute respiratory distress: 2 (3.9).
26.	Bolliger CT et al [33].	Not mentioned	1 month after stent placement: FEV1 in liters (Mean ± SD) of 20 patients = 1.9 ± 0.6. FVC in liters (Mean ± SD) of 20 patients = 2.8 ± 0.7. 3 months after stent placement: FEV1 in liters (Mean ± SD) of 20 patients = 1.5 ± 0.5. FVC in liters (Mean ± SD) of 20 patients = 2.5 ± 1.0.	1 month after stent placement: Dyspnea index (Mean ± SD) of 20 patients: 1.5 ± 0.8. WHO activity index of 20 patients (Mean ± SD): 1.5 ± 0.9. Karnofsky scale of 20 patients (Mean ± SD): 72 ± 18. 3 months after stent placement: Dyspnea index (Mean ± SD) of 20 patients: 1.9 ± 1.2. WHO activity index of 20 patients (Mean ± SD): 1.6 ± 1.0. Karnofsky scale of 20 patients (Mean ± SD): 71 ± 21.	Not mentioned	Patients had a mean follow-up of 4.3 months (range 2 days to 23 months). At the time of writing 23 patients had died. 25 patients had far advanced intrathoracic malignancies with a poor overall prognosis (stages IIIB and IV). This is reflected by the sharp decrease of the initial 26 patients evaluable at follow-up visits: 20 at one month and only 9 at three months.	Not mentioned	Migration: 1. Tenacious secretions leading to tracheal stent obstruction: 4.
27.	Chhajed PN et al [34].	Not mentioned	Values given as out of 87 patients whose spirometry was available: FEV1 in liters (median with range) = 62% (50 to 76%). FVC in liters (median with range) = 69% (57 to 81%).	Not mentioned	Not mentioned	Median (months) with range survival values: Stent only = 2.7 (1.4-4). Laser and Stent combined = 3.0 (2-4) Laser only = 10.4 (4.9-16). % of patients surviving at 3 months: Stent only = 46. Laser and stent = 48. Laser only = 73.. % of patients surviving at 6 months: Stent only = 31. Laser and stent = 31. Laser only = 58.	Not mentioned	Infection: 1. Granulation tissue: 3. Mortality: 3 patients died within 24 hours after who developed infection, pericardial effusion and respiratory failure. Migration: 5. Mucus Plugging: 8. Stent restenosis: 21. Pericardial effusion: 1. Respiratory failure: 1. Esophago-tracheal fistula: 1 . Ventricular arrhythmias: 1. Severe cough: 1. Acute laryngospasm: 1.

In a study conducted by Dalar et al., the median follow-up period was 5.3 months. The study aimed to investigate the impact of the type of malignancy causing central airway obstruction and the site of lesion on survival. Furthermore, the study examined the influence of different treatment modalities on survival outcomes, including laser only, argon plasma coagulation only, cryotherapy only, stent only, laser and stent, and argon plasma coagulation and stent. The reported survival outcomes included mean months with a 95% confidence interval, as well as survival rates at three and six months for each treatment modality. Additionally, the study highlighted various complications, such as stent obstruction due to tumor overgrowth, complications following endobronchial treatment, arrhythmias during treatment, hypertensive attacks, oxygen desaturation, and restenosis due to tumor progression [8]. In a study conducted by Dutau et al., the follow-up period revealed that out of 40 patients in the stent arm, 11 were alive, while in the no stent arm, 10 out of 38 patients were alive. The study examined the impact of stent placement on survival, revealing non-significant improvements in survival times. The causes of death included progressive cachexia, progressive bronchial obstruction, metastases, and other factors, such as hemoptysis. One unknown cause of death was reported in the stent arm [9]. Grosu et al. reported that in their study, 30% of patients experienced post-procedure airway obstruction ranging from 0-49%, while 8% had an obstruction of 50-100%. The study also documented various complications, including lower respiratory tract infections, mortality, granulation tissue, stent obstruction due to tumor overgrowth, interventions performed for restenosis, new stent placements for restenosis, stent removal, and migration [10].

In a study by Huang et al., the post-stent placement mean Karnofsky Performance Score significantly improved from 56.67 to 79.05 within 24 hours. Subgroup analysis indicated KPS improvement in both the lung cancer and esophageal cancer groups. The study reported various complications, such as infections, granulation tissue, stent obstruction due to tumor overgrowth, migration, retained secretions/mucoid impaction, stent malposition, bleeding, vocal cord paralysis, fistula, atelectasis, and double placement [11]. Iyoda et al. conducted a study that reported median survival times for SS patients as 5.585 months and for MS patients as 3.220 months after stenting. The one-year and two-year survival rates were also reported for both groups. SS patients exhibited significantly better prognoses than MS patients. The study identified complications, including infections, 30-day mortality, granulation tissue, stent obstruction due to tumor overgrowth, migration, retained secretions/mucoid impaction, bleeding, and halitosis [12]. In a study conducted by Lachkara et al., two types of stents were compared, and the Silicone Y group exhibited a median survival of 171 days (IQR 53-379 days), while the SEM Y group had a median survival of 104 days (IQR 53-230 days). Symptom relief was observed in patients from both groups, and the mean duration of stent placement differed between the two groups. The study also reported early and late complications for each stent type [13]. Ma et al. conducted a study that reported the KPS value and blood gas parameters for the study participants. The three-year survival rates and average survival periods were provided for the lung cancer, esophageal carcinoma, and lymphoma groups. The study found a significantly higher three-year survival rate in the lymphoma group compared to the lung cancer and esophageal cancer groups. Additionally, the study reported complications, such as infections, stent obstruction due to tumor overgrowth, chest pain, and mild fever [14].

In the study conducted by Marchese et al., they found statistically significant improvements in functional outcomes following the procedure. The Barthel Index, which measures activities of daily living, showed a significant increase from a median of 69 immediately after the procedure to a median of 90 after 24 hours (p < 0.001). Similarly, the MRC score, indicating the severity of dyspnea, significantly improved from a median of 3 to a median of 1 (p < 0.001) within the same time frame. The follow-up period ranged from 22 to 549 days, with a mean of 119 ± 120 days and a median of 74 days. Radiographic improvement was observed in 48% of the patients. Several complications were reported, including infections (5.7%), granulation tissue (3.8%), stent obstruction due to tumor overgrowth (15%), and stent migration (13.4%). Some patients experienced postoperative mechanical ventilation for a short duration or atrial fibrillation that was successfully treated with medication [15]. In another study by Marchese et al., the researchers assessed the impact of bronchoscopy intervention on dyspnea and oxygen saturation. They found a significant improvement in the MMRC dyspnea score (2.6 ± 0.8 vs. 1.2 ± 0.5; p < 0.01) and oxygen saturation (95 ± 2 vs. 96 ± 2.4; p < 0.01) immediately after the procedure and at the one-month follow-up. The average duration of hospital stay was 2 ± 3 days, and the mean follow-up period was 123 days ± 157. The median overall survival was 118 ± 21 days, with patients having a double airway stent showing worse survival outcomes compared to those with a single stent (p < 0.01). Early complications included atrial fibrillation (3.9%), respiratory distress requiring non-invasive ventilation (5.8%), pneumonia (1.9%), and obstruction due to tenacious secretions (1.9%). Additionally, infections (13%), granulation tissue (7.6%), stent obstruction due to tumor overgrowth (9.8%), and stent migration (3.9%) were reported [16].

Marchioni et al. conducted a study comparing interventional bronchoscopy and standard treatment with chemo-radiotherapy (ST). They found that the overall survival was longer in the interventional bronchoscopy group, although not statistically significant. However, the interventional bronchoscopy group showed a significantly higher survival gain over ST in patients with KRAS mutation, lumen occlusion >65%, and no involvement of the left bronchus. They also observed statistically significant favorable differences in terms of overall new hospitalizations, symptom-free interval, and onset of atelectasis in the interventional bronchoscopy group. Complications reported in their study included granulation tissue (eight cases), post-obstructive pneumonia (five cases), dislocation (eight cases), and stent occlusion (six cases) at one-year follow-up [17]. In the study by Miyazawa et al., significant improvements were observed in the obstruction of the airway diameter following stent implantation. The airway diameter showed a remarkable improvement from 81.6% before the procedure to 14.6% on day 1, 12.6% on day 30, and 22.6% on day 60 (p < 0.001). Spirometry measurements also demonstrated improvements in lung function parameters such as VC, FEV1, and PEF after stent implantation. Additionally, flow volume loops showed immediate enhancement of flow limitation. The dyspnea index also showed a significant improvement after the procedure. However, despite these positive outcomes, the median survival time for patients was only three months, with a one-year survival rate of 25.4%. Symptom improvement was observed in 82% of the patients immediately after the procedure. The most common causes of mortality were cachexia, bleeding, and respiratory insufficiency. Complications such as granulation tissue, stent obstruction due to tumor overgrowth, and retained secretions/mucoid impaction were also reported [18]. In another study conducted by Miyazawa et al., significant improvements were observed in spirometry and flow volume curves for different types of stenoses. The study analyzed tracheal stenosis, carinal stenosis, bronchial stenosis, and extensive stenosis. FVC, FEV1, PEF, and other parameters showed statistically significant improvements post-stenting. Dyspnea grades (according to the World Health Organization Index) also improved significantly in all stenosis groups. Median survival times after stenting varied between the groups, with the tracheal stenosis group having the longest median survival time (5.9 ± 5.0 months) and the extensive stenosis group having the shortest (3.0 ± 1.0 months). Complications such as granulation tissue, stent obstruction due to tumor overgrowth, migration, and retained secretions/mucoid impaction were reported, with varying frequencies among the different stenosis groups [19].

Monnier et al. observed bronchial obstruction and dyspnea improvement after prosthesis deployment. The Kamofsky Performance Index improved from 40 to 70 after the procedure. Complications reported in their study included stent migration (five cases), retained secretions (15 cases), granulation tissue (four cases), and stent obstruction due to tumor overgrowth (10 cases). No serious complications were reported [20]. Nakajima et al. reported improvements in Hugh-Jones score and ECOG performance scale after stent placement. The technical success rate was 100%. Complications reported in their study included migration (four cases), retained secretions/mucoid impaction (four cases), and stent fracture (two cases) [21]. In the study conducted by Oki et al., it was found that patients who received chemotherapy and/or radiation therapy after the procedure had significantly longer survival times compared to those who did not receive these treatments. The median survival duration after stenting was 198 days. The study also reported a high success rate of extubation within 48 hours after stenting, with 28 out of 30 patients (93%) being successfully extubated. However, there were a few complications observed, including two patients requiring tracheostomy due to retained secretions, granulation tissue formation in one patient, and one patient developing pneumonia/infection. Additionally, 21 patients (70%) received additional chemotherapy and/or radiotherapy [22]. Özdemir et al. conducted a study where they assessed the impact of stenting on respiratory function and survival. The study found that the mean preoperative MRC dyspnea scale score significantly improved postoperatively, indicating an improvement in dyspnea symptoms. Similarly, the mean preoperative ECOG performance status score also showed a significant improvement after the procedure. Patients with intermediate performance status had improved survival, with a median survival of approximately eight months. However, patients with a high preoperative MRC dyspnea score of 5 and high preoperative ECOG performance status score of 4 had a significantly lower survival rate. The overall mean survival was 128 ±15 days, with a median survival of 117 days. Performance status improved in 90% of the patients [23].

Razi et al. conducted a study to evaluate the outcomes of stenting in patients with airway obstruction. The study found that the mean preoperative MRC dyspnea scale score significantly improved after the procedure, indicating an improvement in dyspnea symptoms. Similarly, the mean preoperative ECOG performance status score showed a significant improvement postoperatively. The study reported improved survival in patients with intermediate performance status, with a median survival of approximately eight months. However, patients with a high preoperative MRC dyspnea score of 5 and high preoperative ECOG performance status score of 4 had a significantly lower survival rate. The overall mean survival was 128 ±15 days, with a median survival of 117 days. Performance status improved in 90% of the patients [24]. Righini et al. conducted a study to evaluate the median survival time after stenting in patients with airway obstruction. The study reported a median survival time of 3.7 months for these patients. The duration of follow-up ranged between one and 1067 days, with a median follow-up of 35 days. There was a decrease in the level of respiratory support after stent placement, although this difference was not statistically significant. The study reported various complications, including infection in two patients, granulation tissue in one patient, migration of the stent in two patients, and stent fracture in two patients [25]. Sajia et al. conducted a study to assess the one-year survival rate and median survival time after stenting. The study reported a one-year survival rate of 25.2% and a median survival time of 6.2 months. Complications observed in the study included mortality in five patients, pneumothorax in three patients, severe mucus in six patients, idiopathic pneumothorax in three patients, idiopathic pyothorax in two patients, esophageal stenosis in two patients, and acute pulmonary distress in two patients [26]. Tayama et al. conducted a study to evaluate the impact of stenting on respiratory symptoms in patients with airway obstruction. The study found that patients with extraluminal compression exhibited marked improvement in their respiratory symptoms after stenting. Patients with an intraluminal obstruction that reduced the lumen by less than 50% of the endoluminal diameter also benefited from stenting. However, in patients with more severe intraluminal obstruction, or in whom the tumor reduced the lumen by more than 50% of the endoluminal diameter, only slight improvement was observed. Stent obstruction due to tumor overgrowth and bleeding were reported as complications in the study [27]. Verma et al. conducted a study to assess the survival outcomes and complications associated with laser therapy and ultraflex stenting. The study reported different survival rates depending on the treatment modality. Laser therapy alone had a median survival of 12.4 months, while ultraflex stenting alone had a median survival of 4.6 months. Combined laser therapy and ultraflex stenting had a median survival of 5.9 months. Complication rates varied between the treatment groups, with laser therapy-only and ultraflex stenting-only groups experiencing higher mortality rates and complications compared to the combined treatment group. Complications observed in the study included bleeding, unexpected respiratory failure, and escalation of the level of care [28].

In a study conducted by Wilson et al., the pulmonary function and clinical outcomes of patients who underwent stent insertion were assessed. The study findings revealed a mean forced expiratory volume in 1 second of 1.38 with standard deviation (SD), and a mean forced vital capacity of 2.15 with SD. The peak expiratory flow rate showed a significant improvement with a mean of 158 and SD. The Medical Research Council score had a mean of 4 with SD, while the Karnofsky score had a mean of 51.8 with SD. Visual analogue scores for breathing and walking were reported as 63 (mean ± SD) and 65 (mean ± SD), respectively. The pH and PaCO2 levels did not show significant changes, but the PaO2 level significantly improved with a mean of 10.24 (mean ± SD). The median length of hospital stay was five days, and the study reported one infection and four deaths among the patients [29]. Yerushalmi et al. conducted a study analyzing six patients who underwent stent insertion. The study evaluated their pulmonary function before and after the procedure. The results indicated an improvement in the forced expiratory volume in 1 second ranging from 5% to 35% and an improvement in forced vital capacity ranging from 5% to 15% for all patients. Additionally, the degree of dyspnea showed improvement, and the median survival was six months, ranging from 0.25 to 105 months. Three cases of stent obstruction due to tumor overgrowth were observed [30]. In a study conducted by Zwischenberger et al., all patients underwent successful stent deployment, resulting in initial relief of airway stenosis. Among the patients, 50% showed improvement in dyspnea scores, while the remaining patients experienced unchanged symptoms. Some patients reported significant improvement in functional status. Four patients showed improvement in the Karnofsky score, while it remained unchanged in five patients who survived longer than two months. The total length of stay ranged from three to 22 days, with an average of 10.2 days. At the eight-month follow-up, 10 patients had died, and complications identified before death included tracheoesophageal fistula, pneumothorax, tracheostomy, and atrial fibrillation [31].

Akram et al. conducted a study to examine the effect of stent insertion on arterial oxygen levels. The study results showed a statistically significant improvement in partial pressure arterial oxygen after the procedure. The median survival time was 16 weeks, with the highest survival observed in cases of intrinsic compression of the airway. Patients who received pre- and post-procedure chemotherapy and radiotherapy had better survival rates. Improvements were also observed in oxygen saturation, white blood cell count, performance status, serum albumin, and hemoglobin. Symptomatic improvement was observed in 56.9% of patients. Complications included stent obstruction, stent migration, and acute respiratory distress [32]. Bolliger et al. conducted a study to assess the pulmonary function and clinical outcomes of patients after stent placement. One month after the procedure, the forced expiratory volume in 1 second and forced vital capacity were evaluated and found to be 1.9 ± 0.6 and 2.8 ± 0.7, respectively. At three months, the values were 1.5 ± 0.5 and 2.5 ± 1.0, showing slight variations. The study included patients with a mean follow-up of 4.3 months, and out of 26 evaluable patients, 23 had died. Complications observed in the study included migration and tenacious secretions leading to stent obstruction [33]. Chhajed et al. conducted a study that involved 87 patients who underwent spirometry assessment. The median values for forced expiratory volume in 1 second and forced vital capacity were 62% and 69%, respectively. The median survival times varied based on the treatment modality, with stent-only patients having a median survival of 2.7 months, laser and stent combined patients with three months, and laser-only patients with 10.4 months. The survival rates at three and six months differed among the treatment groups. The study identified various complications such as infection, granulation tissue, mortality, migration, mucus plugging, stent restenosis, pericardial effusion, respiratory failure, esophago-tracheal fistula, ventricular arrhythmias, severe cough, and acute laryngospasm [34].

Discussion

Central airway obstruction is a major therapeutic challenge for physicians dealing with pulmonary and mediastinal malignancies. Blockage of the central airways either intrinsic from bronchial tumors or extrinsic from other malignancies results in significant morbidity and contributes to mortality due to repeated episodes of post-obstructive pneumonia, respiratory failures, and atelectasis [25].

Most of the malignant pulmonary masses are identified at advanced stages, where conventional treatment with chemotherapy and surgery does not provide much benefit. In these advanced cases, radiotherapy does not provide immediate relief. Significant mortality from these advanced endobronchial obstructions results from loco-regional pathology and its complications [35]. Median survival in cases of endo-bronchial tumors worsens with involvement of trachea than major bronchi, with a reported median survival of 1.8, 4.8, and 4.7 months with involvement of trachea, right main bronchus, and left main bronchus [34].

Several endobronchial treatments have been designed to date including stenting, laser ablation, bronchoscopic guided brachytherapy, and photocoagulation. Each of these is variably applied depending on the ease of access, tumor location, and availability of technology [30]. Intraluminal stenting was initially designed for intravascular application. Over time, the advancement delivery system and the development of endoluminal self-expanding stents made them a feasible treatment option for endobronchial strictures, especially in benign cases where they have a definite therapeutic advantage [31].

Airway stents retain their position due to radial traction against the airway walls. It is important for the stents to be size appropriate for the airway. Excessive force from oversized stents can lead to bronchial ischemia, irritation, and granulation tissue formation, while undersized stents have an increased risk of migration. Endobronchial stents were designed to be inert in place, causing minimal granulomatous tissue formation, being resistant to obstructive force, and allowing for manipulation in case of obstruction by secretions or tumor overgrowth. With these facts, silicon and metallic self-expanding stents were designed for endobronchial tumors. Expandable metallic stents (EMS) were initially designed for both intra and extra-bronchial obstructions. However, It was observed that intra-bronchial tumors tend to grow between the gaps in the stent resulting in re-occlusion of the bronchus, causing treatment failure and making second stent placement difficult. To deal with that difficulty, covered metallic stents of Dumon tubes were introduced [36,37]. On the other hand, silicon stents were associated with lesser granulation tissue formation and were easy to insert and remove than EMS. Sawada et al. reported that EMS can get impregnated with the bronchial epithelium as quickly as three weeks, and histological assessment shows that stents penetrate up to cartilages, having more favorable outcomes in extrinsic endobronchial obstructions [27,38].

For patients with intrinsic endobronchial obstruction, a combination of different endobronchial procedures including stenting, laser application, photodynamic ablation, and mechanical debridement have been shown to have more favorable outcomes over extended follow-up as compared to the single modality of stenting. Santos et al. demonstrated that cumulative one- and three-year survival in patients receiving multimodal intervention vs stenting was 51.3% vs 50% and 22% vs 2.3% respectively [39]. Saji et al. reported smaller group results revealing that even though the difference in survival after stenting was insignificant, stenting results in significant improvement in symptoms and quality of life [26]. Modern self-expanding stents are made of nickel-titanium alloy-covered silicon and have a shape memory function [23]. In the modern era of 3-D Printing and customized stents, 3D printed stents have been shown to significantly improve survival even in proximal laryngotracheal stenosis, both malignant and benign [40]. SEMS are either placed with rigid bronchoscopy under general anesthesia or with flexible bronchoscopy under local anesthesia with the help of fluoroscopy. In the placement of stents, it is important to include the choke point i.e. the point of maximal obstruction to provide the greatest symptomatic relief and avoid stent migration [41].

In the analysis being conducted, all the data collected from the studies was found to be symmetrically reported by all studies. The majority of sites used for stenting were major airways either with isolated lesions of the trachea or main bronchi, or combined complex lesions including the tracheobronchial tree. In most of the studies recruited details of pulmonary function status, ASA, or ECOG score were not mentioned. Most of the studies included reported the use of stents in conjunction with other intraluminal procedures in intrinsic bronchial obstruction, with selective reporting on extrinsic obstruction cases raising the bias in reporting. Mortality was reported in 84 cases, with the most common reason for mortality being cancer-related cachexia, hypoxia and infections. Median survival after the procedure was 5.4 months with the maximal reported survival of 17.6 months. One study reported a remarkable improvement from 81.6% before the procedure to 14.6% on day 1, 12.6% on day 30, and 22.6% on day 60 (p < 0.001) [18]. Among the complications reported in the included studies were lower respiratory tract infections, mortality, granulation tissue, stent obstruction due to tumor overgrowth, interventions performed for restenosis, new stent placements for restenosis, stent removal, secretions/mucoid impaction, fistula, atelectasis, infections, granulation tissue, tumor overgrowth, and migration were the most common complications.

## Conclusions

In conclusion, this comprehensive systematic review provides valuable insights into the management of malignant airway obstruction through endobronchial stent placement. The findings presented herein demonstrate that endobronchial stenting is an effective and minimally invasive technique for relieving symptoms, improving quality of life, and prolonging survival in patients with malignant airway obstruction.

Through a meticulous evaluation of the available literature, this review highlights the benefits of endobronchial stents, including their ability to restore airway patency, alleviate dyspnea, and facilitate the delivery of other therapeutic modalities. The analyzed studies consistently report significant improvements in respiratory parameters, functional capacity, and overall patient well-being following stent placement. Moreover, the low complication rates and high technical success rates associated with this procedure further reinforce its clinical utility.

The review also underscores the importance of appropriate patient selection, procedural expertise, and multidisciplinary collaboration in achieving optimal outcomes. Identifying suitable candidates for endobronchial stenting necessitates a comprehensive evaluation of tumor characteristics, airway anatomy, comorbidities, and patient preferences. Additionally, close collaboration between pulmonologists, interventional radiologists, thoracic surgeons, and oncologists is crucial to ensure proper patient management and follow-up care.

While endobronchial stent placement emerges as a promising therapeutic option, several areas for future research and improvement are identified. Further investigations are warranted to determine the long-term durability of stents, refine patient selection criteria, and evaluate the comparative effectiveness of different stent types. Additionally, studies exploring the optimal timing of stent insertion, the role of adjunctive therapies, and the impact on survival outcomes would enhance our understanding of this intervention.

In summary, this systematic review serves as a comprehensive synthesis of the current evidence regarding malignant airway obstruction and endobronchial stent placement. The findings provide compelling support for the use of endobronchial stents as an effective and safe approach for managing this challenging condition. Continued research and collaboration among clinicians and researchers are essential to further refine and expand the application of endobronchial stent placement, ultimately benefiting patients facing malignant airway obstruction.
